# Mitochondrial dysfunction at the crossroad of cardiovascular diseases and cancer

**DOI:** 10.1186/s12967-023-04498-5

**Published:** 2023-09-19

**Authors:** Carmine Rocca, Teresa Soda, Ernestina Marianna De Francesco, Marco Fiorillo, Francesco Moccia, Giuseppe Viglietto, Tommaso Angelone, Nicola Amodio

**Affiliations:** 1 Cellular and Molecular Cardiovascular Pathophysiology Laboratory, Department of Biology, E and E.S. (DiBEST), University of Calabria, Arcavacata di Rende, 87036 Cosenza, Italy; 2 Department of Health Science, University Magna Graecia of Catanzaro, 88100 Catanzaro, Italy; 3 Endocrinology Unit, Department of Clinical and Experimental Medicine, University of Catania, Garibaldi-Nesima Hospital, 95122 Catania, Italy; 4 Department of Pharmacy, Health and Nutritional Sciences, University of Calabria, 87036 Rende, Italy; 5 Laboratory of General Physiology, Department of Biology and Biotechnology “L. Spallanzani”, University of Pavia, 27100 Pavia, Italy; 6 Department of Experimental and Clinical Medicine, Magna Graecia University of Catanzaro, 88100 Catanzaro, Italy; 7 National Institute of Cardiovascular Research (I.N.R.C.), 40126 Bologna, Italy

**Keywords:** Mitochondrial dynamics, Mitochondrial dysfunction, Cardiovascular diseases, Cancer, Anticancer therapy, Cardiotoxicity

## Abstract

A large body of evidence indicates the existence of a complex pathophysiological relationship between cardiovascular diseases and cancer. Mitochondria are crucial organelles whose optimal activity is determined by quality control systems, which regulate critical cellular events, ranging from intermediary metabolism and calcium signaling to mitochondrial dynamics, cell death and mitophagy. Emerging data indicate that impaired mitochondrial quality control drives myocardial dysfunction occurring in several heart diseases, including cardiac hypertrophy, myocardial infarction, ischaemia/reperfusion damage and metabolic cardiomyopathies. On the other hand, diverse human cancers also dysregulate mitochondrial quality control to promote their initiation and progression, suggesting that modulating mitochondrial homeostasis may represent a promising therapeutic strategy both in cardiology and oncology. In this review, first we briefly introduce the physiological mechanisms underlying the mitochondrial quality control system, and then summarize the current understanding about the impact of dysregulated mitochondrial functions in cardiovascular diseases and cancer. We also discuss key mitochondrial mechanisms underlying the increased risk of cardiovascular complications secondary to the main current anticancer strategies, highlighting the potential of strategies aimed at alleviating mitochondrial impairment-related cardiac dysfunction and tumorigenesis. It is hoped that this summary can provide novel insights into precision medicine approaches to reduce cardiovascular and cancer morbidities and mortalities.

## Introduction

Cardiovascular disorders (CVDs) and cancer represent the two major causes of mortality and morbidity worldwide and, therefore, pose an unprecedented need to identify novel therapeutic treatments to effectively save the patients from death [1]. CVDs and cancer share several common environmental risk factors, such as alcohol abuse, obesity, sedentary life, and tobacco, and improving the lifestyle could be of benefit for patients suffering from these diseases [2]. Furthermore, the same genetic and cellular mechanisms could play a pathogenic role in both CVDs and cancer, including inflammatory signalling, reactive oxygen species (ROS), Wnt signalling pathway, and hypoxia-inducible factor-1α (HIF-1α) [1, 3–5]. In addition, the incidence of CVDs is significantly higher in cancer patients, and cancer survivors undergo a higher risk to develop CVDs [6], such as chronic heart failure [7]. Therefore, new diagnoses of malignancy in subjects with pre-existing HF can often occur, requiring a complex and early integrated cardio-oncological approach for their clinical management. The concept that cancer represents a risk factor for CVDs, such as heart disease, atherosclerosis, cerebrovascular disease, hypertension, and aortic aneurysm/dissection, is increasingly being recognized. Accordingly, cancer may directly affect cardiac function as evinced by the increased medium-term to long-term risk for one or more CVDs showed in survivors of most site-specific cancers compared with that for the general population [8].The site of cancer also represents an important factor in determining the risk of CVDs in cancer survivors. In this regard, a recent Surveillance, Epidemiology, and End Results program-based observational study in US population, which represents the largest study on the deaths from CVDs among patients with 28 cancer types with over 40 years of follow-up, reported that cancer patients exhibit an on average 2–6 times higher CVDs mortality risk than the general population, with the majority of deaths from CVDs occurring in patients presenting with breast, prostate, or bladder cancer. Conversely, patients with endometrial cancer showed a very high risk of dying from CVDs at the first year after diagnosis, which remains elevated compared to other cancer sites [9]. Cardiotoxicity, in turn, is a recognized side effect of anticancer treatments that can ultimately lead to therapy discontinuation in some oncological patients [10–12].

On this evidence, cardio-oncology has emerged as a common framework for cardiologists, oncologists, and hematologists to identify and target the most adverse cardiovascular events that prevent cancer patients to benefit from a wide variety of therapeutic approaches [13, 14].

Mitochondria are dynamic organelles that not only constitute the cell powerhouse by producing most of the chemical energy (adenosine-5-trisphosphate, ATP) that supports cellular activities through oxidative phosphorylation (OXPHOS) and fatty acid oxidation, but also serve as a signalling hub by regulating a plethora of cellular processes, including ROS generation and maintenance, phospholipid biosynthesis, intracellular Ca^2+^ regulation, angiogenesis, innate immune signalling, and iron-sulphur cluster biosynthesis [15–20]. Mitochondria are no longer regarded as static and passive sub-cellular structures [21]; they are now considered dynamic organelles that can change shape, structure, number, and intracellular positioning through a finely tuned regulatory network, known as quality control system, which fulfills the primary function to meet ATP supply with the high-energy demand of vital organs, such as heart and brain [15, 22–25]. Defects in mitochondrial function, as well as mitochondrial dynamics and quality control, have been reported in both CVDs and cancer, which present deregulated bioenergetics and metabolism [11, 26–28] and show a dramatic deregulation of mitochondrial-derived intracellular signals, such as ROS and Ca^2+^ [16, 27, 29]. In addition, mitochondria sense and respond to stressors signals, such as DNA damage and hypoxia, which is a hallmark of multiple disorders, including CVDs and cancer [4, 11, 30]. Finally, several anticancer therapies cause life-threatening cardiovascular complications by inducing mitochondrial stress or dysfunction, which may also initiate many secondary, systemic signalling pathways that further exacerbate cardiovascular damage [11]. On this basis, herein we first survey the molecular mechanisms which regulate mitochondrial dynamics and quality control and illustrate how they can be altered both in CVDs and cancer. In parallel, we delineate the emerging therapeutic avenues that aim at reducing the burden of mitochondrial damage in those regarded as the most life-threatening disorders worldwide. Finally, we highlight the cardiovascular complications deriving from anti-cancer therapies, and illustrate how mitochondria-targeted strategies could improve the therapeutic outcome of cancer patients without causing cardiotoxicity.

## Mitochondrial physiology: focus on mitochondrial dynamics and quality control systems

Mitochondria are highly dynamic organelles that can adapt to different conditions by reversibly cycling through two distinct morphological states—a short rod-like shape and an interconnected pattern—by dividing and fusing the inner and outer mitochondrial membranes (IMM and OMM, respectively). Mitochondrial dynamics refers to the dynamic behaviour of mitochondria, including the fusion and fission of mitochondrial membranes [31]. Such fission–fusion cycle is intricately regulated by a highly conserved mechanism involving a group of proteins that are located on the IMM and OMM and belong to the dynamin family of GTPases. Mitofusin 1 and 2 (MFN1/2) and optic atrophy 1 (OPA1) are responsible for fusion, while dynamin-related protein 1 (DRP1) and dynamin-2 (DNM2) are the primary fission proteins [32–34].

### Mitochondrial fusion

Mitochondrial fusion is the process by which two mitochondria combine to form one mitochondrion. A typical mitochondrial fusion reaction involves two mitochondria colliding end-to-end; the membrane fusion event occurs at the site of the collision. Fusion reactions can also occur end-to-side or even within a single mitochondrion to form structures that resemble rings. Because mitochondria have two sets of membranes, the fusion process begins with the outer membrane and continues with the inner membrane. These two membrane fusion events occur almost simultaneously, although a temporal distinction can sometimes be identified. OPA1 and MFN1/2 are responsible for triggering the IMM and OMM fusion processes during mitochondrial fusion [31]. Although the primary function of OPA1 is to control mitochondrial fusion, it also has unrelated activities, such as improving mitochondrial respiratory efficiency, stabilizing the structure of mitochondrial cristae, controlling the release of cytochrome c, and triggering the assembly of electron transport supercomplexes [35]. MFN2 has activities unrelated to nuclear fusion. Specifically, MFN2 strengthens mitochondrial calcium buffering by connecting mitochondria and the endoplasmic reticulum (ER) [36]. Phosphorylated MFN2 acts as a mitochondrial receptor for the protein Parkin and mediates mitophagy in cardiomyocytes [37]. The function of OPA1 is controlled not only by the cleavage of OPA1 by proteases but also by post-translational modifications. In cardiac mitochondria, there are two forms of OPA1: the long form of OPA1 (L-OPA1), which binds to the IMM, and the soluble short form of OPA1 (S-OPA1), which is located in the intermembrane space representing a product of L-OPA1 proteolytic cleavage. The presence of both L-OPA1 and S-OPA1 in mitochondria facilitates the process of mitochondrial fusion [38]. In humans, L-OPA1 has two distinct cleavage sites. The S1 site, found in all forms of L-OPA1, is cleaved by the protease OMA1, and the S2 site, only found in some forms of L-OPA1, is cleaved by YME1L. These two cleavage points are distinct from one another [39]. In most cases, YME1L acts as a mediator for the proteolysis of L-OPA1 at the S2 site. This process produces S-OPA1 and unsevered L-OPA1, both of which promote mitochondrial fusion and maintain cristae morphology. When cells are stressed, however, OMA1 activity increases, and OPA1 undergoes proteolysis at the S1 site. This causes all forms of L-OPA1 to be cleaved into S-OPA1, which causes mitochondrial fragmentation, cristae morphology disruption, and apoptosis [40]. OMA1 activation and L-OPA1 processing may be linked to the proapoptotic signalling pathways, and this activation may be dependent on the oligomerization of BAK and BAX [41]. The idea that elevated S-OPA1 levels and OMA1 activity impair mitochondrial and cardiac function was disproven by the work of Lee et al., who demonstrated that increasing S-OPA1 levels improved cell survival under oxidative stress by preserving mitochondrial cristae and energetics. The suppression of OMA1 expression leads to more oxidative stress-induced cell death, suggesting that OMA1-induced S-OPA1 production represents a survival mechanism in stressed cells [42]. Alongside proteolysis, post-translational modifications are another mechanism for controlling OPA1 function. Sirtuin 3, a mitochondrial deacetylase, protects cardiomyocytes from doxorubicin-induced cell death by increasing OPA1’s GTPase activity through deacetylation of OPA1 at Lys 926 and Lys 931 [43]. In addition, OPA1 was found to be O-GlcNAcylated in the presence of hyperglycemia, which reduces its GTPase activity and ultimately leads to cardiomyocyte death [44]. Like OPA1, MFN1/2 can be regulated through post-translational modifications. Cells are particularly susceptible to apoptotic stimuli when MFN1 is phosphorylated by ERK at T562, which reduces its efficiency in oligomerization and mitochondrial tethering but increases its binding to BAK [45]. In addition to upregulating the activity of protein kinase C beta II (PKC beta II), phosphorylation of MFN1 at Ser86 reduces MFN1 GTPase activity and leads to an increase in the accumulation of fragmented and dysfunctional mitochondria, both of which are associated with HF [46]. The phosphorylation of MFN1 appears to reduce its GTPase activity, which in turn reduces mitochondrial fusion and impairs mitochondrial function. Increased mitophagy and decreased accumulation of mitochondria with abnormal structure and function are achieved through PTEN-induced putative kinase 1 (PINK1)-mediated phosphorylation of MFN2, which in turn promotes Parkin translocation to mitochondria [47, 48]. Previous studies indicated that the protective effect of Notch1 on cardiomyocytes under myocardial reperfusion injury is associated with a decrease in MFN2 phosphorylation [49]. OPA1 has an important role in maintaining cardiac health and function. The homozygous mutation of OPA1 is lethal in mice, and even heterozygous OPA1^+/-^ mice show significant cardiac dysfunction by the age of 12 months [50]. The overexpression of OPA1 encourages the assembly of respiratory chain supercomplexes (RCS) and the formation of tight cristae, in contrast to its conditional ablation, which compromises cristae integrity and slows respiratory function and growth [51].

### Mitochondrial fission

Mitochondrial fission is the process by which a mitochondrion fragmentates into smaller mitochondria. DRP1 is a GTP-hydrolyzing enzyme that plays a crucial role in mediating mitochondrial fission [52, 53]. DRP1 and its receptors—mitochondrial fission protein 1 (FIS1), mitochondrial dynamics proteins of 49 and 51 kilodaltons (Mid49/51), and mitochondrial fission factor (MFF)—regulate mitochondrial fission [54]. The DRP1 protein is divided into four structural domains: the GTPase domain, which generates mechanical force; the self-assembly domain; the GTPase effector domain, which regulates the amount of GTPase activity; and the variable domain (B-insert), which mediates interactions between proteins. Phosphorylation, SUMOylation, and O-GlcNAcylation represent some examples of the plethora of post-translational modifications that have been linked to the B-insert gene [55]. Stress-induced alterations in the post-translational structure of DRP1, its translocation to the OMM, and its binding to receptors at INF2-marked precontraction sites, all contribute to the cellular response known as mitochondrial contraction. During this process, DRP1 oligomerizes and forms ring-like multimeric structures that resemble those of dynamin rings. In a GTP-dependent process, DRP1 allows the mitochondrion to divide into two separate mitochondria. DRP1 activation and mitochondrial fission are both influenced by posttranscriptional modifications, particularly phosphorylation and dephosphorylation, as well as SUMOylation and deSUMOylation. Ser616, Ser637, and Ser656 phosphorylation sites in DRP1 protein have been extensively studied and characterized [53, 56].

### Ubiquitin proteasome system (UPS) and mitochondrial unfolded protein response (UPR.^mt^)

Typically, mislocalized or damaged proteins are degraded by the ubiquitin–proteasome system (UPS) in the cytosol [57, 58]. Proteases, such as CLPP and LONP1, are responsible for the degradation of damaged or misfolded proteins inside the mitochondria, while chaperones, such as HSP10, HSP60, and DNAJ, are responsible for ensuring that newly imported proteins fold properly. In response to mitochondrial stresses, such as oxidative stress, ischemia/reperfusion (I/R) injury (IRI), impairment of mitochondrial DNA or metabolic function, and/or perturbation of mitochondrial proteostasis, the mitochondrial unfolded protein response (UPR^mt^) is activated [59–61]. Particularly, UPR^mt^ represents an adaptive program that controls mitochondrial homeostasis and reduces unfolded and misfolded protein amounts, thanks to its adaptive ability to respond to stressful conditions by promoting the transcription of genes encoding mitochondrial chaperones and proteases. This intricate quality control mechanism, also involved in sensing defects in mitochondrial translation and OXPHOS biogenesis, contributes to combat mitochondrial stress and dysfunction, exerting pleiotropic actions for maintaining cellular homeostasis [62–65]. The UPR^mt^ is widely accepted as the first line of defense against mitochondrial damage. When stress levels rise above a certain threshold, mitophagy is triggered and removes dysfunctional mitochondria. The expression of UPR^mt^ genes is regulated in mammals by the activating transcription factor 5 (ATF5), which contains a basic leucine zipper (bZIP) that binds to mitochondrial targeting sequences (MTSs) and nuclear localization sequences (NLSs). Under physiological conditions, ATF5 is degraded by the protease LON, which specifically targets ATF5 in the mitochondrial matrix [66–68]. When mitochondrial proteostasis is disrupted, ATF5 activates genes encoding for UPR^mt^-associated proteins [63]. In addition to ATF5, ATF4 and CHOP also play a role in UPR^mt^ activation [63, 69]. How ATF4, CHOP, and ATF5 all contribute to UPR^mt^ activation is currently not completely deciphered. However, the integrated stress response (ISR), which involves the regulation of protein biosynthesis in response to mitochondrial damage, is connected to these three transcription factors [63, 70]. By pharmacologically stimulating the ISR while blood flow through coronary arteries is restored, IRI can be prevented [71]. The primary regulator of the ISR, i.e., eIF2, is activated by the mitochondrial stress transmitter axis OMA1/DELE1/HRI [72]. Increased cardiomyocyte survival, decreased ATP demand, and reduced ROS production after IRI, are all results of eIF2 activation [73, 74]. After eIF2 activation, expression of the transcription factors ATF4, ATF5, and CHOP is enhanced, leading to increased expression of chaperones and proteases [75, 76]. In mammals, UPR^mt^ activation correlates with the induction of the ISR and eIF2. However, additional investigation aimed at improving the knowledge regarding the role of mitochondrial dysfunction, the UPR^mt^, and the ISR in cardiac IRI is required.

### Mitophagy

Unnecessary or broken cell components must be removed from all cells. The accumulation of toxic waste, the accommodation of new elements, and the reuse of old building blocks are all unfeasible without the complex system of autophagy [77, 78], subclassified in macroautophagy, microautophagy, and chaperone-mediated autophagy (CMA). To engulf and transport cargo to the lysosome, macroautophagy requires autophagosome formation. The direct translocation of unfolded proteins across the lysosomal membrane is facilitated by CMA. In microautophagy, smaller waste products are directly engulfed in the lysosomal matrix through invaginations of the lysosomal membrane [78, 79]. The best-characterized pathway for mitochondrial turnover, i.e., macroautophagy—henceforth referred to as autophagy—involves the degradation of large cellular components or even organelles, including mitochondria (mitophagy).

Mitophagy is responsible for removing damaged or redundant mitochondria. Mitophagy is crucial to the quality control system of the mitochondria being an integral component of the mitochondrial response to stressor inputs [66, 80]. As a consequence, when mitophagy is impaired, mitochondria do not work or there is an accumulation of mitochondria in the cytoplasm, which can alter cell homeostasis and lead to diseases. Two main mechanisms of mitophagy have been identified: ubiquitin-mediated mitophagy and receptor-mediated mitophagy, which may, respectively, occur through the PINK1-Parkin-mediated ubiquitin pathway and the FUN14 domain-containing protein 1 (FUNDC1) receptor-mediated pathway [81, 82]. Some essential mitochondrial proteins are ubiquitylated in PINK1-Parkin mediated regulation of mitophagy. The kinase PINK1 is first synthesized in the cytoplasm and subsequently translocates into the IMM, in which it is targeted to the ubiquitin-mediated proteasome pathway upon cleavage by PARL protease [83]. Selective accumulation of the active PINK1 in OMM occurs when PINK1 degradation is inhibited because of mitochondrial impairment or loss of mitochondrial membrane potential (ΔΨm) [84]. OMM-localized PINK1 homodimerizes, auto-phosphorylates, and phosphorylates ubiquitin (Ub) at Ser65. The phospho-Ser65-Ub, in turn, recruits cytosolic Parkin to the OMM, where it is phosphorylated by PINK1 at Ser65 and can thereby initiate its E3 ubiquitin ligase activity [85]. Parkin is thus able to ubiquitinate multiple targets that are located on the outer mitochondrial surface, including voltage-dependent anion channel (VDAC), MIRO1, and MFN1/2 [86]. Notably, MFN2 phosphorylation by Parkin is instrumental to prevent the fusion between dysfunctional and healthy mitochondria [37]. Autophagy receptors, such as Sequestosome 1 (p62/SQSTM1), optineurin (*OPTN)*, neighbor of BRCA1 gene 1 (*NBR1*), and coiled-coil domain-containing protein 2 (NDP52), recognize the ubiquitinated mitochondrial proteins and link them to LC3-II for autophagic degradation. LC3-II binds the autophagosomal membrane to autophagy proteins (ATG), and together drive the OMM to fuse with lysosomes, thereby forming the autolysosomes. Finally, cathepsins and lipases degrade the autophagosome content removing damage mitochondria [87, 88].

Mitophagy may also occur in a ubiquitin-independent manner, which does not involve Parkin, but is mediated by mitophagic receptors that are expressed on the mitochondrial outer membrane [89, 90]. These receptors include FUNDC1 [91], as well as the Bcl-2 interacting protein 3 (BNIP3) and its analogue NIX [92]. The crucial component of these receptors is a conserved LC3-interacting region (LIR) that binds to and connects LC3 to the mitochondrial outer surface. FUNDC1 phosphorylation at Tyr18 by receptor-interacting serine/threonine kinase 3 (RIPK3) and SRC prevents the binding of the LIR domain to LC3-II. However, hypoxia, which represents a common hallmark of both CVDs and cancer, prevents FUNDC1 phosphorylation and thus enables LC3-II recognition and binding to FUNDC1, thereby targeting the mitochondria for mitophagy [81, 82]. Similarly, BNIP3 and NIX expression is increased by hypoxia and regulated by HIF-1α. However, they also belong to the pro-apoptotic BH3-only proteins and can also participate in OMM permeabilization, mitochondrial permeability transition pore (mPTP) opening and cytochrome C release. Therefore, their role in mitophagy needs to be further investigated [81, 82].

## Mitochondrial dysfunction in cardiovascular diseases (CVDs)

The progressive decline of mitochondrial function occurring during CVDs is associated with alterations in the respiratory chain and ATP synthesis, excessive ROS production, and structural abnormality of mitochondria. These processes lead to cell damage and cardiomyocyte death occurring via apoptosis, triggered by cytochrome c release, or necrosis, induced by mPTP opening [93, 94]. Accordingly, it is widely accepted that inhibiting mPTP blunts the loss of cardiac myocytes driving numerous heart diseases, including myocardial IRI, diverse cardiomyopathies, and the cardiotoxic actions of anti-cancer agents [95]. Although there are no large, randomized studies assessing the therapeutic potential of strategies aimed at preventing/relieving mitochondrial dysfunction in CVDs, numerous pre-clinical researches have been conducted in this field highlighting mitochondrial function as a promising target for the treatment of these pathologic states, that represent a major health problem worldwide, as well as the main cause of death in the Western world [96, 97]. Indeed, it is widely accepted that maintaining mitochondrial function and integrity is a major factor for the physiological functions of cells and their survival, particularly for non-dividing cells endowed with a high-energy demand and producing high levels of oxidative stress, such as cardiomyocytes [96, 97]. Therefore, it is imperative to ensure and coordinate important quality control mechanisms in cardiomyocytes, taking part to mitochondrial biogenesis, mitochondrial dynamics, and mitophagy, to prevent mitochondrial dysfunction and the progression of CVDs [98].

### Imbalance of mitochondrial dynamics and mitophagy

**Fig. 1:**
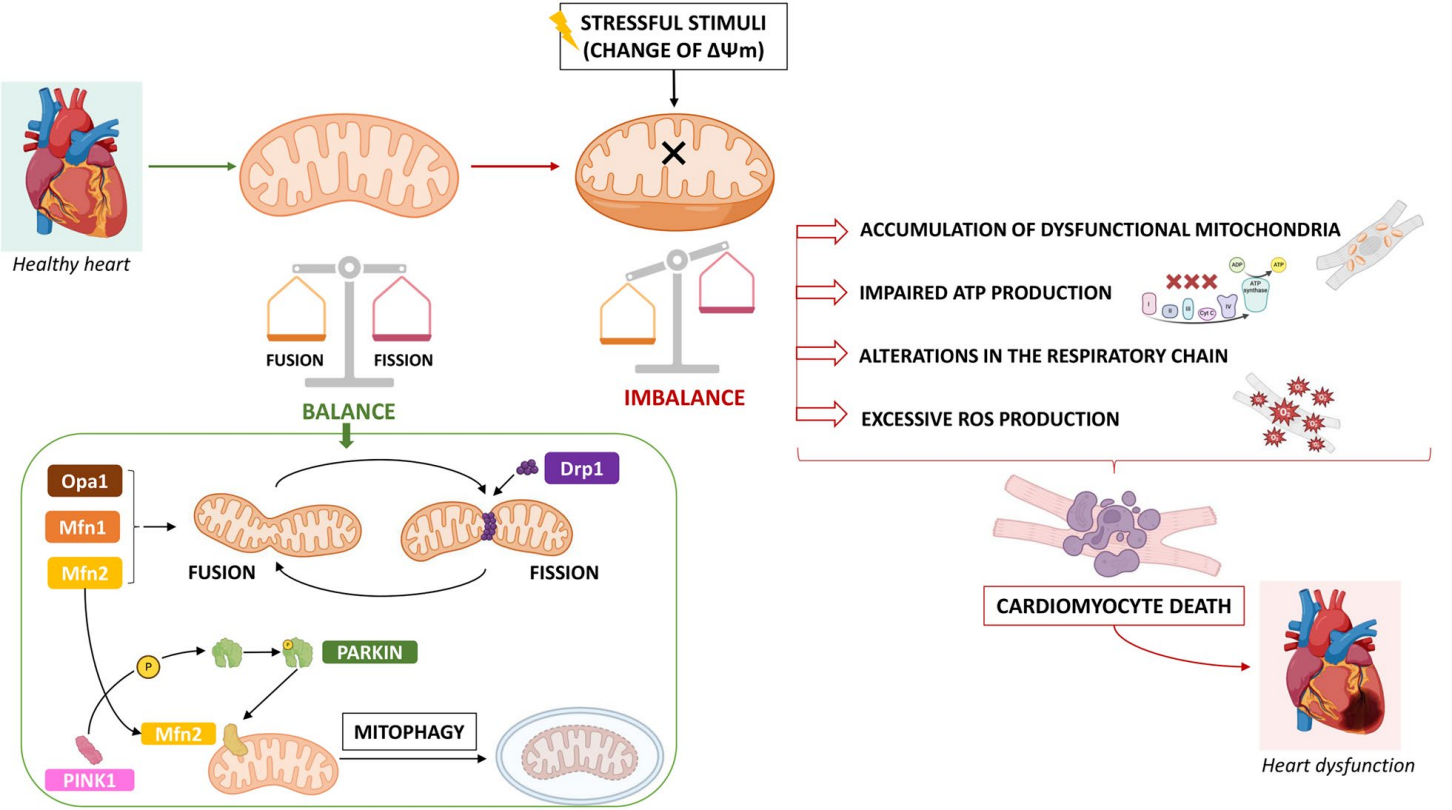
General representation illustrating the consequences of the imbalance of mitochondrial dynamics, culminating in heart disease. See text for details. *Drp1* dynamin-related protein 1, *Mfn1* mitofusin 1, *Mfn2* mitofusin 2, *OPA1* optic atrophy 1, *PINK1* PTEN-induced putative kinase 1, *ΔΨm* mitochondrial membrane potential

An emerging body of pre-clinical evidence indicates that alterations of intrinsic quality control mechanisms, including fusion, fission, and mitochondrial autophagy, are implicated in the progression of numerous CVDs (Fig. 1). As a matter of fact, a central link exists between the dysregulated mitochondrial dynamics and mitophagy, the accumulation of dysfunctional mitochondria and impaired ATP production, leading to mitochondrial dysfunction and cardiac disease. Recent reports also indicate that key metabolic changes drive pathological cardiac remodelling, and mitochondrial dynamic appears fundamental for the correct balance between energy demand and nutrient supply, suggesting that changes in mitochondrial morphology may act as a mechanism for bioenergetic adaptation during HF [99].

One of the reason behind the association between alteration of mitochondrial dynamics and numerous CVDs lies in the fact that mitochondrial dynamics often represents the first response against mitochondrial stress, such as changes in ΔΨm, and this is particularly true for cardiomyocytes, since mitochondria are especially abundant in cardiac tissue, representing approximately 30% of the total cell volume, and since cardiomyocytes require high levels of ATP to support cardiac function [100].

Given the fundamental role of mitophagy, as an autophagic response targeting damaged, dysfunctional, and cytotoxic cardiac mitochondria, it can be assumed that disturbed mitophagy can cause mitochondrial damage and ultimately cardiomyocyte death. Direct evidence comes from genetic and pharmacological studies in rodent models of CVDs, indicating that altering autophagy or mitophagy, for example by limiting autophagic or mitophagic flux at the whole-body level or in specific compartments of the cardiovascular system, can increase the propensity to spontaneously develop cardiodegenerative disorders or influence disease severity in several cardiovascular conditions, including ischemic heart disease, cardiomyopathies, and atherosclerosis. In this regard, Bravo-San Pedro et al. provided important mechanistic details, highlighting the cardiovascular consequences of deleting autophagy- and mitophagy-relevant genes in the heart, including but not limited to *Atg5* (autophagy related 5), *Bnip3l* (BCL2 interacting protein 3 like), *Dnml1* (dynamin 1 like), *Lamp2* (lysosomal-associated membrane protein 2), *Mfn1*, *Mfn2,* the proautophagic transcription factor *FOXO3* (forkhead box O3), *Park2* [Parkinson disease (autosomal recessive, juvenile) 2], parkin, and Pink1 (PTEN-induced putative kinase 1) [101]. Although the detailed molecular mechanisms by which disrupted mitophagy leads to mitochondrial damage and cardiomyocyte death, driving the progression of CVDs, are largely unknown, a direct link has been established between mitochondrial damage secondary to cardiovascular stress and the exacerbation of the imbalance of autophagy or mitophagy (excessive inhibition or promotion). This indicates that preserving autophagic/mitophagic responses is crucial to safeguard the cardiovascular homeostasis and prevent premature degenerative changes in cardiovascular tissues. For instance, in coronary heart disease/acute MI or IRI, where increasing studies are delineating the role of FUNDC1-, PINK/Parkin-, and BNIP3-dependent mitophagy, these pathways represent potential important targets to improve myocardial IRI (see the recent review by Liu and Mu for further details) [102]. Other studies reported the protective role of mitophagy during IRI and other numerous CVDs, including diabetic cardiomyopathy, HF, hypertension, arrhythmia, stroke, valvular dysfunction, and aging (see the recent review by Yang et al. for further details) [103].

Collectively, if many studies indicated mitophagy as a key protective program for mitochondrial function and cardiomyocyte homeostasis, other experimental findings reported that mitophagy may also play a negative role in heart disease, such as IRI and HF. Indeed, ischemic/hypoxic environment and/or the excess of ROS (i.e., during myocardial reperfusion) may activate mitophagy through BNIP3, Beclin-1 and ATG5, impacting ATP production, and exacerbating myocardial injury. A “mitophagy paradox” has also been hypothesized in IRI, since mitophagy seems to be protective (adaptive) during acute ischemic phase, where it contributes in removing dysfunctional mitochondria and preserving basal metabolic requirements, while the excessive mitochondrial fission, fragmentation and mitophagy (maladaptive) may induce unnecessary mitochondrial clearance and impair mitochondrial integrity and function during the reperfusion phase [104].

Noteworthy, the role of mitophagy during IRI appears even more complex if we consider that mitophagy also acts as “mediator” during inflammatory damage of cardiomyocytes or vascular endothelial cells to remove inflammation-induced damaged mitochondria under normal conditions [102]. It is known that inflammation plays a central role in the early stages of coronary heart disease, driving the formation of atherosclerotic plaque or myocardial fiber plaque [105]. Impaired mitophagy also associates with inflammation, particularly through the activation of NLRP3 inflammasome, which in turn can be responsible for endothelial damage and increased accumulation of cholesterol in macrophages [106]. Additionally, a defective mitophagy in mice lacking *Fundc1*, a newly characterized mitophagy receptor, is associated with metabolic disorders via MAPK signaling and inflammatory responses [107]. Conversely, a very recent study reported that tumor necrosis factor receptor-associated factor-2 (TRAF2), an innate immunity effector, is essential for physiological mitophagy in cardiomyocytes, since its myocardial loss (in inducible cardiac-myocyte specific TRAF2 *knockout* mice) impairs physiological mitophagy in the heart inducing cardiac inflammation, thus supporting a cardioprotective role of TRAF2-stimulated mitophagy [108].

On the other hand, an uncontrolled activation of mitophagy can lead to the accumulation of damaged mitochondria and inflammatory response activation, reduction of myocytes, and contractile impairment, which ultimately contribute to cardiac aging and HF [99]. Other evidence indicates that excessive mitophagy leads to mitochondrial population decline, impaired oxidative phosphorylation, and decreased ATP production, culminating in HF [109, 110]. According to these reports, in the failing heart, the stress-induced mitophagy acts as a maladaptive response to hemodynamic parameters such as pressure overload inducing a negative remodeling of the myocardium, mainly mediated by Beclin-1 [111, 112]. Overall, given the complex and multifaceted involvment of mitophagy in the regulation of myocardial injury, it should be considered that the protective or damaging role in activating mitophagy needs to be contextualized to the degree and duration of cell stress, the different stages of myocardial injury, as well as the degree of mitophagy that, physiologically can be beneficial, while at excessive or inadequate levels can be deleterious.

#### Implication of the fission protein DRP1 in CVDs

Direct evidence attesting the crucial implication of mitochondrial dynamics in CVDs derives from studies reporting how alterations in the role of endogenous factors, that physiologically regulate mitochondrial fission and fusion, can dramatically participate to the onset and progression of cardiac dysfunction. For instance, mitochondrial fission abnormalities generated by Drp1 disruption may induce mitochondrial elongation and inhibit mitochondrial autophagy, thereby resulting in mitochondrial dysfunction and promoting cardiac dysfunction and increased susceptibility to IRI in mice [113]. Similarly, downregulation of mitochondrial autophagy can induce mitochondrial dysfunction and HF, while its restoration mitigated the progression of HF in a mouse model of pressure overload, where endogenous Drp1 was crucial for mediating mitochondrial autophagy and maintaining both mitochondrial and cardiac function [114]. Another study indicated that Drp1 inhibition significantly contributed to the accumulation of altered cardiac mitochondria by repressing BNIP3-induced mitophagy, suggesting that that Drp1-mediated mitochondrial fission is a prerequisite for mitophagy during cardiac hypertrophy [56, 115].

The essential role of endogenous Drp1 was further demonstrated in adult mouse cardiomyocytes with ablated Drp1, that caused dramatic alterations in mitochondrial fission and promoted mitophagic mitochondrial depletion, contributing to the lethal cardiomyopathy [116]. In support of this, the homozygous deletion of Drp1 in mice is responsible for embryonic lethality, which is due to elongated mitochondria, altered apoptosis and reduced cell proliferation, while postnatal cardiac specific knock out of Drp1 leads to dilated cardiomyopathy and rapid lethality in mice [117, 118]. Based on these knowledge, the role of Drp1 in the pathogenesis of several CVDs, including pulmonary arterial hypertension, HF, cardiac hypertrophy, IRI and myocardial infarction (MI), has been extensively addressed in the last years, highlighting the potentiality of this factor to effectively represent a strategy to treat CVDs [56].

**Fig. 2:**
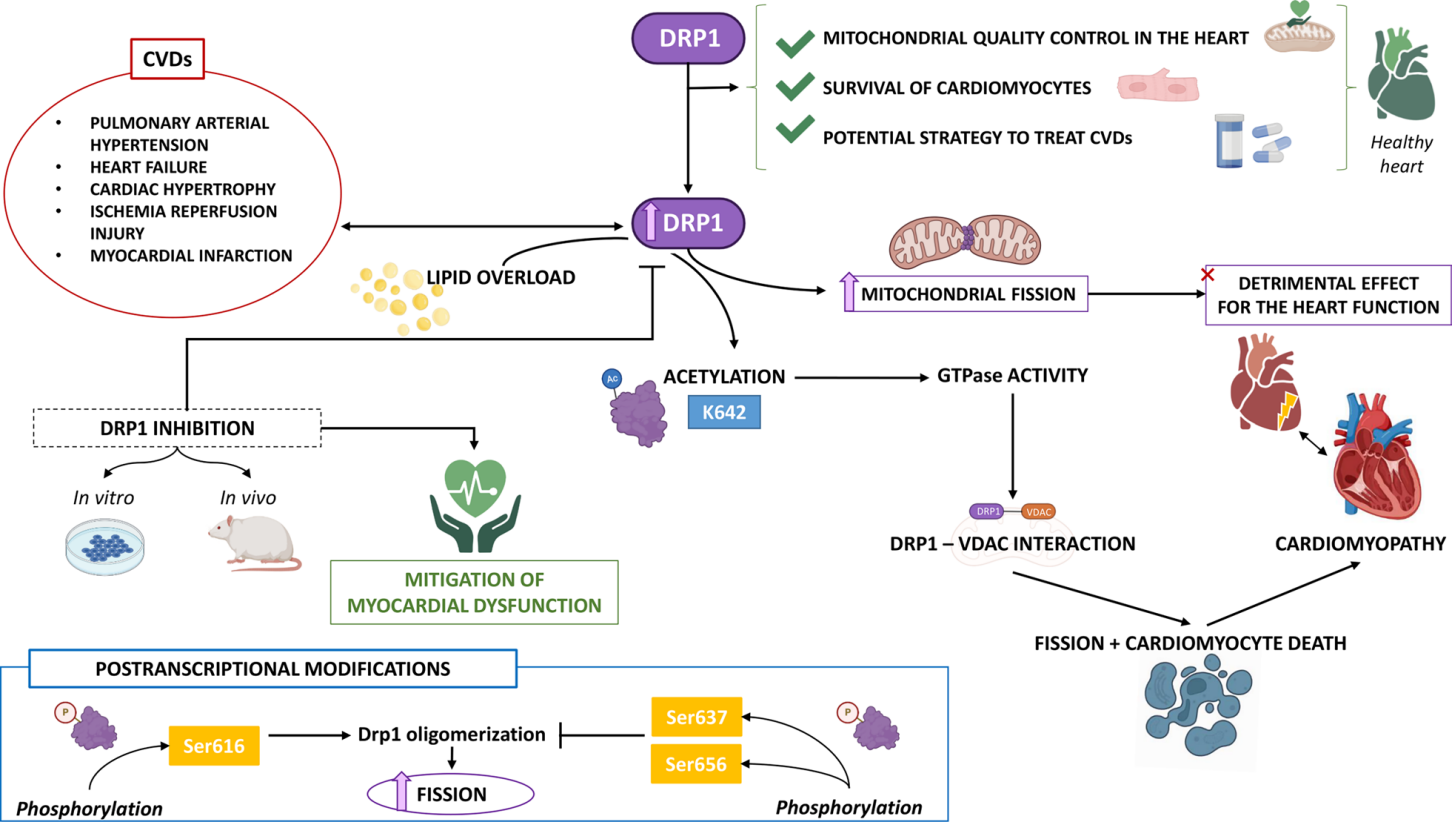
Proposed mechanisms underlying the detrimental effects deriving from an imbalanced mitochondrial dynamic in the heart, with particular regard to the implication of Drp1, a protein playing a fundamental role in heart physiology and cardiomyocyte survival during normal conditions, but that it is upregulated during cardiac pathological conditions, resulting in excessive fission. See text for details. *CVDs* cardiovascular diseases, *Drp1* dynamin-related protein 1, *VDAC* voltage-dependent anion channel

However, Drp1 upregulation has also been reported in several CVDs, where the excessive fission of mitochondria impairs the cardiac function (Fig. 2). For instance, a dominant-negative mutant form of Drp1 decreased mitochondrial fission, mPTP sensitivity, and cell death in cardiac-derived HL-1 cells subjected to IRI; a similar cardioprotective effect was obtained by inhibiting Drp1 with the Mitochondrial division inhibitor-1 (Mdivi-1) in HL-1 and mice exposed to coronary artery occlusion and reperfusion [119]. Additional reports confirmed the cardioprotective action of Drp1 inhibition against IRI in rat and mouse models by preserving mitochondrial function and reducing cell death [120–123]. Drp1 inhibition was also effective in relieving myocardial dysfunction in in vitro and in vivo models of insulin resistance and metabolic cardiomyopathy, as well as cardiac hypertrophy and HF [56, 100].

Focusing on the very recent studies, Hu et al. provided novel evidence about the mechanism by which Drp1 can contribute to the lipid overload-induced heart hypertrophy and dysfunction through modulation of the intracellular redox environment [124]. In particular, by using high-fat diet (HFD)-induced metabolic dysfunction in mouse and monkey models, and in vitro palmitate model, the authors found that decreased NAD^+^ levels and increased of Drp1 acetylation at Lys642 occur. The consequent excessive Drp1 activation through phosphorylation, mitochondrial translocation, and oligomerization, showed higher GTPase activity, bound with voltage-dependent anion channel 1 (VDAC1) on mitochondria, to induce mitochondrial fission and cardiomyocyte death; this suggests that acetylation represents a novel post-translational Drp1 modification that regulates its activity, contributing to metabolic cardiomyopathy (Fig. 2).

Drp1 activation and mitochondrial fission are influenced by additional specific posttranscriptional modifications. In particular, Ser616 phosphorylation stimulates Drp1 oligomerization and mitochondrial fission by targeting Drp1 to the OMM [125]; conversely, Ser637 and Ser656 phosphorylation blocks Drp1 oligomerization and prevents mitochondrial fission [126, 127]. Ser616 phosphorylation is upregulated in response to IRI via either the activation of CDK1, PKC, PGAM5, ROCK, and GSK-3 or the inhibition of PKC [128]. IRI injury also promotes Ser637 dephosphorylation, which activates Drp1 and promotes mitochondrial fission [129]. Increased cardiac PIM-1 expression prevents cell death in response to IRI by increasing Drp1 phosphorylation at Ser637 [121]. PKA phosphorylates Ser656, while calcineurin dephosphorylates it [127, 130]. Nitrite-mediated cardiomyocyte protection against IRI has been linked to PKA activation, which inhibits Drp1 activity [130]. MiR-199 prevents calcineurin from phosphorylating Drp1 during MI, which prevents apoptosis [127]. SUMOylation by SUMO-1 promotes Drp1 localization to mitochondria, while SUMOylation by SUMO2/3 inhibits it [131]. SUMOylation can be degraded in response to the activity of SENP families [132]. A decrease in SUMO-2/3-modified Drp1 and an increase in mitochondrial fission are caused by increased SENP5 expression following cardiac IRI [133]. SENP3 exacerbates cardiac IRI by facilitating Drp1 translocation to mitochondria during reperfusion [134]. MFF undergoes posttranscriptional modifications in the same way that Drp1 does. Succinate induces MFF phosphorylation in a GPR91-dependent fashion during myocardial ischemic injury. In turn, MFF activation promotes mitochondrial fission after ERK1/2 activation [135]. Furthermore, NR4A1 expression is upregulated after cardiac microvascular reperfusion injury; this upregulation promotes MFF phosphorylation by activating CK2a, leading to fatal mitochondrial fission [136]. Myocardial reperfusion damage is characterized by an increase in MFF phosphorylation via activation of the JNK pathway and a heightened rate of mitochondrial fission [137]. It is unclear, however, how other posttranscriptional modifications of receptors may influence mitochondrial fission in myocardial reperfusion damage. The production of numerous daughter mitochondria, which in turn supply cardiomyocytes with ATP, is the result of efficient mitochondrial fission. In addition, damaged mitochondria cannot be removed during mitophagy without the help of a process called mitochondrial supply [138]. Particularly, the increased phosphorylation of the kinase JNK during IRI in mice activates MFF and Bnip3, that contribute to the fatal mitochondrial fission and mitophagy, respectively via the caspase 9-related apoptosis and mitochondrial metabolism alterations. The role of MFF during microcirculatory IRI has been deciphered using homozygous Mff-deficient (Mff^gt^) mice that, compared with the wild type counterpart, showed smaller infarct size, restored cardiac function, improved blood flow, and reduced microcirculatory perfusion alterations [139]. This study also provides evidence on the critical contribution of MFF-dependent mitochondrial fission in mediating the microcirculatory IRI through VDAC1/hexokinase 2-mediated mPTP opening, mitochondrial ROS overproduction, and cardiolipin oxidation-involved in cytochrome c leakage into the cytoplasm. Other studies reported an important implication of MFF-dependent mitochondrial division in the development of HF. In this regard, Chen et al. demonstrated that *Mff* mutant mice exhibit, in addition to increased mitophagy, reduced mitochondrial density and respiratory chain activity, and died at 13 weeks due to a severe dilated cardiomyopathy leading to HF [140].

In line with these results, a recent study contributed in deciphering the implications of pathological fission and mitochondrial failure in sepsis-induced cardiomyopathy, focusing on the role of Drp1/FIS1 (mitochondrial adaptor fission 1 interaction of Drp1) interaction. P110, an adaptor-specific peptide inhibitor of Drp1/FIS1 interaction, was found to inhibit lipopolysaccharide (LPS)-induced oxidative stress and mitochondrial fragmentation in H9c2 cells and *Balb/c* mice, which presented an improved cardiac function and reduced mortality. These data indicate that Drp1/FIS1-mediated mitochondrial fission represents an important mechanism of cardiac dysfunction during sepsis [141]. On the other hand, Sun et al. further elucidated the role of Drp1 during IRI in human and mouse models by indicating the beneficial contribution of the TBC domain family member 15 (TBC1D15) in the regulation of mitochondrial homeostasis through the interaction with Drp1 at the mitochondria–lysosome contacts [142]. This report highlights that TBC1D15 may also serve as a potential regulator of mitochondrial homeostasis in the heart and during IRI, suggesting its combination with Drp1 to support asymmetrical mitochondrial fission and clearance for preserving mitochondrial integrity.

#### Involvement of the mitochondrial fusion mitofusin 2 (Mfn2) and optic atrophy 1 (OPA1) in CVDs

The specific cardiac deletion of Mfn2 leads to cardiac hypertrophy with systolic dysfunction [37, 143]. On the other hand, it has been demonstrated that Mfn2 prevents the excessive accumulation of autophagosomes during the IRI phase of the cell cycle in the heart and mediates autophagosome fusion with lysosomes [144]. The conditional combined ablation of Mfn1/Mfn2 in adult hearts induced mitochondrial fragmentation, cardiomyocyte and mitochondrial respiratory dysfunctions, and rapidly progressive and lethal dilated cardiomyopathy (DCM) [145]. In vitro findings obtained in HL-1 cardiac cells and neonatal rat ventricular myocytes (NRVMs) also demonstrated that the Mitofusins may have a cardioprotective role against cardiac dysfunction and IRI, since the overexpression of Mfn1 or Mfn2 can mitigate cell death, while knockdown of Mfn2 can worsen cell survival following acute IRI [119, 146]. Other evidence indicates that Mfn2 ablation reduces mitochondrial Ca^2+^ overload, interrupts fatal Drp1-induced mitochondrial fission and PINK1/Parkin-induced mitophagy, and reduces some of the damage caused by low oxygen levels in cardiac microvascular endothelial cells (CMEC) [147]. These effects, however, are highly dependent on the amount of Mfn2 present. In this regard, in the case of Mfn2 overexpression, Mfn2 facilitates the process by which mitochondria adhere to the sarcoplasmic reticulum (SR). As a result, there is an excess of free Ca^2+^ in the mitochondria, which ultimately leads to mitochondrial damage during IRI [148]. In addition, the hearts of adult mice with combined acute ablation of both Mfn1 and Mfn2*,* despite apparent mitochondrial dysfunction, were protected from in vivo acute MI and IRI; in particular, the cardioprotective phenotype of Mfn1 and Mfn2 depletion was due to beneficial effects on mitochondrial Ca^2+^ levels, oxidative stress, and mPTP opening [146, 149]. These contrasting results may depend on the pleiotropic actions of the Mitofusin proteins [150], as well as on the experimental/clinical context [151]; indeed, given the crucial role of Mfn2 in tethering SR and mitochondria to ensure the physiological inter-organellar Ca^2+^ signaling in the myocardium [152], the reduced interaction between mitochondria and SR during IRI reduces mitochondrial Ca^2+^ overload, resulting in cardioprotection [149]. These findings suggest that the role of Mitofusins in tethering the SR to mitochondria is more relevant for affording cardioprotection against acute IRI than changes in mitochondrial morphology per se. The conclusions of this study were further confirmed by Seidlmayer et al. [153], who generated cardio-specific tamoxifen-inducible Mfn2 KO mice to show that, when the physical linkage between SR and mitochondria by Mfn2 is disrupted, inositol 1,4,5-trisphosphate (IP_3_)-mediated SR Ca^2+^ release fails to induce ATP production. These findings may explain the decreased mitochondrial network excitability in HF cardiomyocytes, caused by disrupted mitochondrial network ultrastructure and impaired mitochondrial functional tethering, accompanied by decreased Mfn1 and Mfn2 levels [154].

On the other hand, several studies reported decreased levels of fusion proteins, including Mfn2 and OPA1, in failing hearts of small and large pre-clinical models of CVDs and patients with HF. In particular, depending on the aetiology and time course of hypertrophy, Mfn2 was downregulated in phenylephrine treated NRVMs, as well as and in hypertrophied failing hearts of spontaneously hypertensive rats and mice with pressure-overload hypertrophy. These findings establish a causal relationship between Mfn2 and cardiac hypertrophy [155]. Consistently, Sabbah et al. observed decreased levels of fusion-associated proteins (Mfn2 and OPA-1) concomitant to increased levels of fission-associated proteins (Fis-1 and Drp1) in left ventricular tissue from dogs and humans with HF (idiopathic dilated cardiomyopathy or ischemic cardiomyopathy), that were reverted by a long-term therapy with elamipretide (ELAM), a water-soluble tetrapeptide targeting mitochondrial dynamics [156]. Decreased levels of Mfn1 were also reported in cardiac tissues of HF patients presenting with idiopathic DCM who do not respond to established multidisciplinary treatment and associate with poor outcomes, that correlated with increased mitochondrial fragmentation [157]. As elegantly reviewed in [143], although additional studies are required for improving the knowledge regarding the involvement of Mitofusin proteins in cardiac hypertrophy and HF, enhancing Mfn2 function may be useful to obtain a therapeutic effect.

Similarly, the protein levels of OPA1 were decreased in rat and human ischaemic HF, and in H9c2 cells exposed to ischemia; on the other hand, reducing OPA1 through shRNA increased mitochondrial fragmentation and decreased tubularity of the mitochondria [158]. The crucial implication of OPA1 was confirmed by the lethal embryonic phenotype following its complete ablation resulting in small and fragmented mitochondria and altered fusion [158, 159], while OPA1^+/–^ mice exhibited late-onset (12 months) left ventricular dysfunction associated with impaired mitochondrial function [50]. Reductions in OPA1 expression and mitochondrial respiratory complex downregulation have been found in hearts damaged by reperfusion. Reducing myocardial injury through drug pretreatment or OPA1 overexpression improves mitochondrial function and decreases mitochondrial fission and expression of the mitochondrial respiratory complex [160]. In reperfused heart tissue, OPA1 deletion contributes to increase the severity of mitochondrial fragmentation and cell damage [161]. Also in this case, additional studies in humans are required to extend the knowledge on OPA1 regulation to move towards the development of novel therapies for OPA1-specific cardiac disease progression [162].

Overall, these findings demonstrate that maintaining an adequate balance between mitochondrial fusion and fission, as well as an optimal mitophagy, is determinant for mitochondrial morphology and cardiac physiology and that, following mitochondrial stress, fusion and fission represent a key response to preserve a healthy mitochondrial network. The effects of fusion and fission are dependent by the magnitude of activation of each process and their balance, as well as by the molecular actors participating in their modulation. This may likely explain their adaptive or maladaptive response following cardiovascular stress [163]. However, the mechanisms by which mitochondrial fusion and fission machineries are altered during CVDs and how this disruption exactly promotes pathological states need to be further explored. The current scenario suggests that a decrease in fusion process, together with increased Drp1 activation, can shift the balance towards fission process, leading to excessive mitochondrial fission and favouring the onset and development of CVDs [164].

Another aspect that should not be underestimated, and that further proves the key role of balanced fusion and fission of mitochondria for an optimal cardiac function, regards the importance of a balanced OPA1 processing, regulated by the two mitochondrial proteases, OMA1 and the AAA protease YME1L. As demonstrated by Wai et al. [165], L-OPA1 is required for mitochondrial fusion and preserves cardiac function. The authors found that the cardiac-specific ablation of Yme1l can activate OMA1 inducing OPA1 proteolysis, resulting in mitochondrial fragmentation, altered cardiac metabolism and HF. On the other hand, cardiac function and mitochondrial morphology were restored by Oma1 deletion, which prevents OPA1 cleavage and mitochondrial fragmentation. Interestingly, this study also showed that mitochondrial fragmentation was accompanied by a metabolic switch from fatty acid to glucose utilization in the heart, a condition that was reverted by metabolic interventions able to preserve cardiac function despite the presence of mitochondrial fragmentation [165]. These findings not only demonstrate the crucial role of OPA1 processing for mitochondrial and heart function, but also suggest that the metabolic switch in the substrate utilization occurring in the failing adult heart may represent a maladaptive process, as previously reported [94].

Nan et al. demonstrated that epigallocatechin gallate, acting as an OMA1 inhibitor, protects cardiomyocytes from IRI damage by reducing OPA1 cleavage; OMA1 activity and S-OPA1 levels are important targets to attenuate myocardial IRI [166]. However, the mechanism that triggers their activation in response to myocardial damage is still unclear. Kent and colleagues found that neither ROS scavengers nor mPTP inhibitors play a causal role in OMA1 activation, even though both increase cardiac and mitochondrial function [167]. Jang et al. demonstrated that high Ca^2+^-mediated mitochondrial swelling facilitates L-OPA1 cleavage in cardiac mitochondria [168]. YME1L deletion in cardiomyocytes can also be triggered by activating OMA1 and triggering L-OPA1 proteolysis [165].

### UPR^mt^ in heart diseases

The involvement of UPR^mt^ in cardiac pathophysiology has been widely investigated and, although there is an important consensus about its adaptive cardioprotective role in diverse CVDs (including IRI, chronic hemodynamic overload and HF), some studies suggested that UPR^mt^ may promote the progression of heart diseases, since blocking several UPR^mt^ elements can reduce the signs of HF of a different aetiology.

Smyrnias et al. found an activated UPR^mt^ in isolated cardiac myocytes exposed to different stressful stimuli and in mice subjected to chronic pressure overload, while pharmacologically boosting UPR^mt^ using nicotinamide riboside significantly reduced cardiac dysfunction [169]. In the hearts of patients with aortic stenosis, who often show left ventricular pressure overload, they also observed an increased expression of UPR^mt^-associated genes, that correlated with a reduction of cardiac dysfunction-related biomarkers (*i.e.*, high-sensitivity troponin T and N-terminal pro–B-type natriuretic peptide) [169]. These results indicate the importance of UPR^mt^ stimulation to promote cardioprotection, providing important evidence that UPR^mt^ may serve as an adaptive process. Xu and collaborators demonstrated that choline can attenuate the mito-nuclear protein imbalance and that the activation of UPR^mt^ is fundamental for choline-mediated cardioprotection, thereby preserving the ultrastructure and function of mitochondria during cardiac hypertrophy [170]. According to these findings, other recent evidence confirmed the cytoprotective significance of UPR^mt^ signalling following mitochondrial dysfunction in heart diseases. In particular, the UPR^mt^ inducers oligomycin or doxycycline protected the heart against IRI in ex vivo and in vivo settings in a mechanism strictly requiring ATF5, since UPR^mt^ induced cardioprotection in wild-type but not ATF5-deficient mice, providing first evidence about the fundamental action of ATF5 for the effectiveness of mammalian UPR^mt^ in vivo [171]. In addition, Zhang et al. have demonstrated that ATF5 is a downstream effector of PGC-1. UPR^mt^ activation via the PGC-1/ATF5 axis mediates a cardioprotective effect in pathological cardiac hypertrophy [172]. On the other hand, by using both gain- and loss-of-function mouse models, together with isolated NRVMs, Venkatesh et al. demonstrated that LonP1 (i.e., an essential mitochondrial protease and component of UPR^mt^ with key roles in maintaining mitochondrial proteostasis and mitigating cell stress) preserves mitochondrial redox status and reduces both oxidative protein damage and cardiomyocyte apoptosis during IRI by reducing Complex I activity [173]. Another investigation indicated that, in a rat model of neurogenic hypertension induced by intracerebroventricular infusion of angiotensin II, enhancing sympatho-excitation can block miR-18a-5p/HIF-1α signaling and increase mitochondrial stress proteotoxicity, reflected by decreased UPR^mt^ and mitochondrial dynamics/OXPHOS/ΔΨm, and oxidative stress. These findings indicate that the consequent mitochondrial abnormalities drive pathological cardiac remodeling culminating in cardiomyopathy [174].

There is also evidence that UPR^mt^ might be associated with harmful events in the heart. For instance, the localization of the mitochondrial chaperone molecule heat shock protein 60 (HSP60) to the cell surface triggered the innate immune system, inducing the release of tumor necrosis factor-α (TNF-α), which correlated with increased myocyte apoptosis in a rat coronary ligation model of HF. The excessive mitochondrial activation of Omi/HtrA2, a mitochondrial serine protease involved in mitochondrial homeostasis, whose levels are increased during UPR^mt^, decreased Δψm, induced cytochrome c release into cytosol through its serine protease activity, and promoted apoptosis in the hearts of aged rats [175]. Additionally, transgenic mice overexpressing cardiac-specific mitochondrial Omi/HtrA2 showed increased myocardial apoptosis and decreased systolic and diastolic function; these effects were prevented by Ucf-101, a specific Omi/HtrA2 inhibitor [125]. Other studies reported increased levels of numerous factors participating to the UPR^mt^ and exerting maladaptive roles, in pre-clinical and clinical models of HF [176–178].

Overall, these studies suggest that the degree of UPR^mt^ activation represents an important element in determining the beneficial or the detrimental cardiac effect of UPR^mt^. In particular, as an adaptive response, UPR^mt^ induces a fundamental cardioprotective response following different cardiac stressful conditions when is moderately activated; under these conditions, UPR^mt^ ensures the optimal control of damaged mitochondrial proteins and maintains the mitochondrial and cardiac functions. Conversely, when excessively activated, UPR^mt^ may be responsible for an exaggerated cleavage of mitochondrial proteins, resulting in a cardiotoxic response and exacerbating cardiac dysfunction. Moreover, in examining the adaptive or maladaptive role of UPR^mt^ during heart diseases, the different experimental settings, the type and duration of stressful conditions, as well as the complex aetiology and the multifactorial aspects of these pathologies, should be considered. This may provide other possible explanations behind the conflicting results on the dual (cardioprotective/cardiotoxic) role of UPR^mt^ in the heart.

Noteworthy, these studies also indicate that endogenous activation of UPR^mt^ appeared to be not sufficient to completely prevent myocardial injury, since the administration of UPR^mt^ activators seems necessary to further improve UPR^mt^ activity and afford additional cardioprotective action. Although other studies are required for explaining this mechanism, it should be noted that UPR^mt^ is also involved in the removal of dysfunctional mitochondria by mitophagy. Indeed, the misfolding of mitochondrial proteins, that are critical for mitochondrial function, induces mitophagy and UPR^mt^ co-activation, highlighting the importance of UPR^mt^ as a regulator of mitochondrial quality control in the heart and indicating that myocardial stress activates endogenous UPR^mt^ and mitophagy that work together to sustain mitochondrial performance and cardiac function [157]. The relationship between mitophagy and UPR^mt^ was also recently demonstrated in a pre-clinical model of septic cardiomyopathy; here, the authors observed that mitophagy activation represents a direct consequence to the fact that endogenous UPR^mt^ cannot completely repair mitochondrial damage under stress conditions; on the other hand, they also suggest that UPR^mt^ may act as a compensatory mechanism in response to mitophagy repression [179].

### Mitochondrial DNA mutations and CVDs

Mitochondrial dysfunctions leading to CVDs may also be due to mutations in either mitochondrial genome (mtDNA) or nuclear genome (nDNA). A primary risk factor for CVDs is indeed represented by mtDNA mutations, which can lead to disruption of mitochondrial homeostasis, oxidative stress, OXPHOS impairment, and energy metabolism damage. Therefore, genetic investigation of mtDNA mutation may be a useful therapeutic approach to detect and forecast CVDs [180]. mtDNA is a circular, double-stranded genome that of 16,569 base pairs in length and contains 37 genes necessary for aerobic respiration and the synthesis of cellular energy via the OXPHOS pathway. Since mtDNA is not recombined and is not protected by histones, its mutation rate is roughly 10–100-fold higher than nuclear DNA [181]. Mutations of mtDNA, which can occur as point mutations, deletions, fragment deletions, or large-scale mtDNA rearrangements, may directly impair OXPHOS [182]. Many cardiac diseases have been linked to mutations in mtDNA [183]. For instance, cardiomyopathy has been linked to mtDNA mutations in mitochondrial DNA, which supports the idea that proteins encoded by mtDNA are crucial to mitochondrial function in the heart [184]. HF may also be regarded as a bioenergetic disorder characterized by extensive mutations in mtDNA as well as by mitochondrial dysfunction, as outlined above [185]. The clinical manifestation of infantile cardiomyopathy is linked to the MRPL44-disorder, which disrupts the translation of a partial protein involved in OXPHOS [186], 187. mtDNA and functional integrity cannot be preserved in cardiomyocytes with myocardial hypertrophy [188] due to abnormal mitochondrial structure and dysfunction of mitophagy clearance. In nearly 40% of patients [189], hypertrophic cardiomyopathy is the most common form of cardiomyopathy associated with mtDNA diseases. Maternally inherited essential hypertension (MIEH) has been linked to mtDNA mutations in a number of studies [182, 190] suggesting that these changes may be one of the pathological mechanisms underlying MIEH. Furthermore, atherosclerosis is linked to mtDNA mutations [191]. The MT-RNR1 gene mutation m.A1555 G, the MT-TL1 gene mutation m.C3256 T, the MT-TL2 gene mutation m.G12315A, and the MT-CYB gene mutation m.G15059A have all been linked to atherosclerosis [192]. A crucial determinant of CVDs may also be represented by mtDNA mutations leading to OXPHOS impairment [96]. In individuals with CHF, it has been observed that a decrease in succinyl-CoA levels within myocardial mitochondrial leads to a reduction in OXPHOS [193]. In a recent analysis of samples harvested from human thoracic aortic aneurysm tissues, the suppression of mitochondrial OXPHOS-related gene expression resulted in an increase in chromatin OXPHOS-related genes. However, despite this increase, the production of ATP remained inadequate to sustain the contractile activity in human aortic smooth muscle cells (HAoSMCs) [194]. In a separate study examining the impact of NOTCH1 deletion on the contractile phenotype and mitochondrial dynamics of human HAoSMCs, it was observed that NOTCH1 deficiency can lead to mitochondrial dysfunction in HAoSMCs, reflected by a decrease in mitochondrial fusion, resulting in the loss of ΔΨm, an increase in ROS generation, inadequate ATP production, and an associated impairment in the contractile phenotype [195]. The deficiency of PGC-1β in the heart leads to the suppression of gene expression related to OXPHOS. This deficiency has the potential to hinder the progression from pressure overload-induced myocardial hypertrophy to HF by regulating the activity of PGC-1β [196].

## Mitochondrial dysfunction in cancer: mechanisms and therapeutic targeting

### Mitochondrial dynamics in cancer

Malignant transformation associated with cancer development has been associated with abnormal mitochondrial dynamics [197–199]. Mitochondria exist along fragmented and fused states regulating mitochondrial metabolism and cell death, and loss of proper mitochondrial dynamics impinges on cell growth and survival [200, 201]. Dysregulated expression ratio of Drp1/MFN1-2 proteins, triggered by multiple genetic or epigenetic events [202, 203] has been found to associate with enhanced cell proliferation and metastatic behaviour [204], as well as to poor prognosis in many cancer types [205]. Mitochondrial fission mediated by ROS imbalanced production represents a primary reason of hepatocellular carcinoma cell survival [73]. Mitochondrial fission also promotes chemotherapy resistance in numerous cancer types [206]. Damaged mitochondria are removed through mitophagy via an increase in mitochondrial fission in healthy cells, while enhanced mitochondrial fission results in OXPHOS defects, cellular and mitochondrial dysfunctions, thereby supporting high proliferation and invasiveness [207]. In several preclinical studies, fission-regulating proteins have become a functional target to restore the mitochondrial network observed in healthy cells, emerging as biomarker of therapeutic efficacy [208]. Indeed, DRP1 inhibitors appear useful anti-cancer therapeutics [208] targeting the survival, apoptosis, and drug resistance of breast cancer cells [204]. For instance, the DRP1 inhibitor Mdivi-1, as well as the novel ellipticine derivative Dripitor1a, decrease oxidative metabolism in cancer and promote cell growth inhibition in vitro and in vivo [209]. In prostate cancer cells, the Drp1-partner MFF was found to interact with the voltage-dependent anion channel-1 (VDAC1) at the OMM, and blocking such interaction led to a collapse of mitochondrial functions with increased MOMP, loss of inner membrane potential, Ca^2+^ unbalance, bioenergetics defects, and activation of cell death [210]. Worthy of note, targeting MFF–VDAC1 complex via an MFF peptidomimetic was safe towards health cells while triggering anti-cancer activity in several preclinical models [211, 212].

**Fig. 3:**
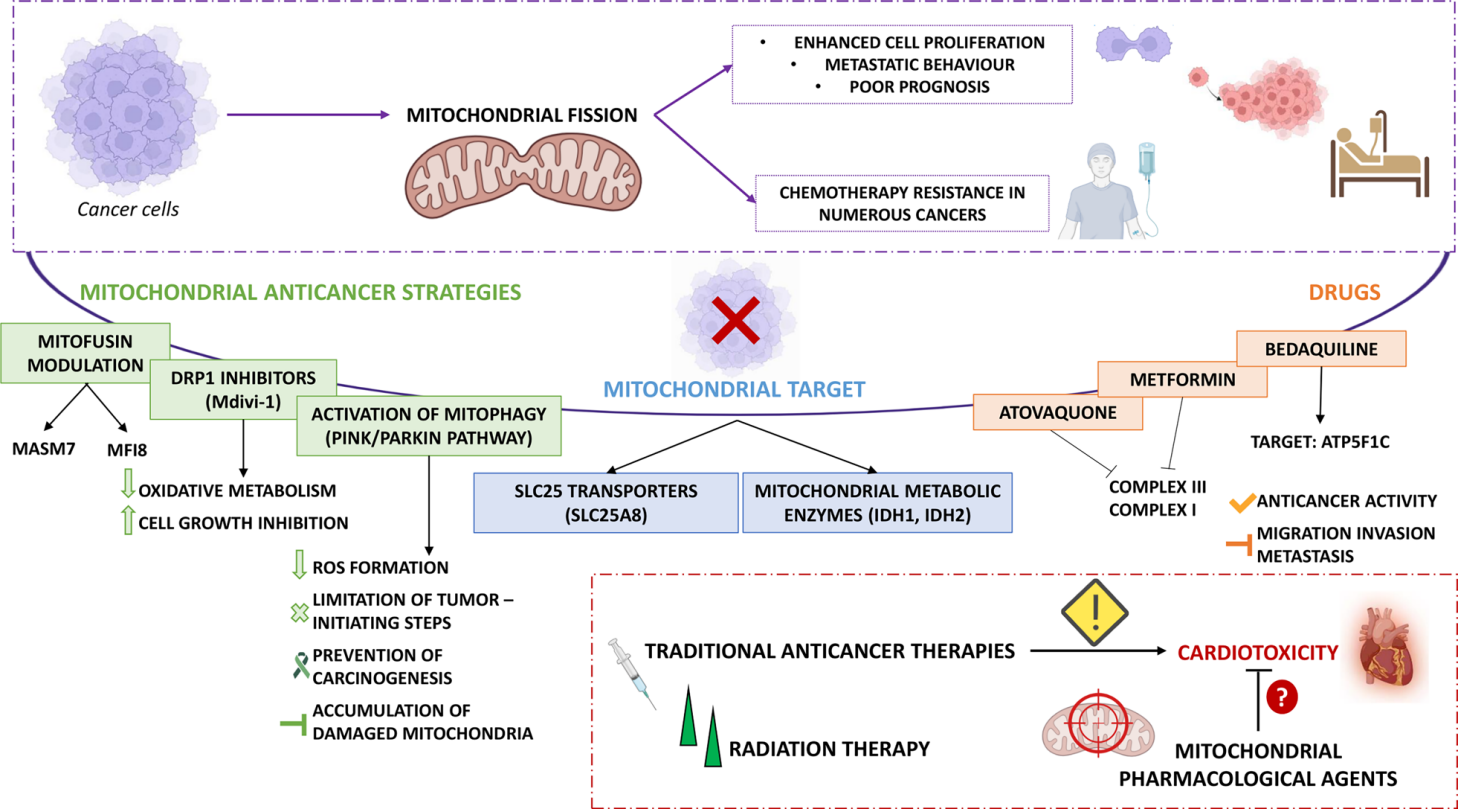
Schematic representation of dysregulated mitochondrial dynamics in cancer suggesting that mitochondrial-dependent mechanisms may serve as potential pharmacological/therapeutic target aimed at restoring mitochondrial network and function. A brief representation is provided for the central role of mitochondria in the adverse cardiovascular events following anticancer therapies. See text for details. *ATP5F1C* ATP synthase F1 subunit gamma, *Drp1* dynamin-related protein 1, *IDH1* isocitrate dehydrogenase [NADP(+] 1, *IDH2* isocitrate dehydrogenase [NADP(+)] 2, *MASM7* mitofusin activator small molecules 7, *Mdivi-1* mitochondrial division inhibitor-1, *MFI8* mitochondrial fusion inhibitor 8, *SLC25A8* UCP2 (uncoupling protein 2)

Mitofusin modulation could also represent a promising anticancer strategy. Recently, it has been shown that the mitofusin activator small molecules 7 (MASM7) induces mitochondrial fusion increasing ΔΨm, mitochondrial respiration, and ATP production. Conversely, mitochondrial fusion inhibitor 8 (MFI8) induces mitochondria fragmentation decreasing ΔΨm, mitochondrial respiration, and ATP production [213] (Fig. 3).

Numerous drugs such as 4-bromo-resveratrol, nicotinamide, sirtinol, cambinol, salermide induce mitochondrial impairment through the inhibition of sirtuins which are directly involved in ATP production [214, 215]. These deacetylases play a key role not only as regulators in many metabolic processes such as glycolysis, OXPHOS, oxidative stress, but they are also implicated in many diseases, including cancer [216]. Indeed, SIRT3 activates the complex I of the electron transport chain, as well as directly regulates the peroxisome proliferator-activated receptor-gamma coactivator PGC1α alongside ERRα under oxidative stress conditions [43, 217]. PGC-1/ERRα axis controls mitochondrial OXPHOS activity and it has been reported as a new pathway driving tumor progression and resistance [218]. In accordance, Peroxisome proliferator-activated receptor gamma coactivator 1-alpha (PGC-1α), the master regulator of mitogenesis, finely regulates mitochondrial dynamics through autophagy and mitophagy [219, 220]; SIRT3 and PGC1α are also correlated by the activation of the AMPK signalling pathway, which increases PGC1α gene-expression [221]. PGC1/NRF1 gene signature have been shown to predict tumor recurrence, metastasis and poor overall survival in both estrogen receptor (ER +)/Luminal-A breast cancer patients [222].

Mitochondrial dynamics has been reported to influence survival and stemness features of cancer stem cells (CSCs) [222, 223], a small sub-population of cancer cells resistant to most anti-cancer therapies, and responsible for tumor recurrence, metastatic dissemination, treatment failure and poor clinical outcomes in nearly all cancer patients [224, 225]. In this context, PGC1α is necessary for the transcription of nuclear-encoded mitochondrial genes promoting mitochondrial biogenesis that is also required for the anchorage-independent survival and propagation of CSCs [226, 227].

### UPR^mt^ in cancer

The UPR^mt^ plays a crucial role in cancer cell survival, anticancer drugs resistance and is emerging as a new therapeutic target [228, 229]. UPR activation has been reported in many human cancers, although it remains partially characterized [230, 231]. Several studies showed a dual role of the UPR in cancer, without a clear definition of the pro-survival and the pro-death effects [229]. Activation of the cytoprotective UPR signalling can promote tumorigenesis and resistance to oxidative and therapeutic stress [232]. Moreover, the UPR^mt^ cellular response is generated by an unbalance between nuclear DNA-encoded OXPHOS proteins and mtDNA-encoded OXPHOS proteins [233]. The UPR^mt^ stimulates mitochondrial protective genes and ROS defence antioxidants making a homeostatic activation within the mitochondrial protein-folding environment [234]. Indeed, UPR^mt^ intricately interfaces with stress response pathways which drive autophagy and mitophagy acting as co-factors also in Sirtuin signalling [235, 236]. Moreover, UPR^mt^ is activated during the disruption of electron transport balance of OXPHOS complexes that are encoded by both mitochondrial and nuclear genomes [237]. Downregulation of complex IV, complex III and ubiquinone synthesis also induces UPR^mt^ [237]. Recent studies reported that the overexpression of misfolded proteins in the mitochondrial intermembrane space, promotes an additional UPR^mt^ axis regulated by the estrogen receptor alpha (ERα) in cancer. The ERα- UPR^mt^ axis is initiated by ROS, which in turn activate Akt, thereby leading to the phosphorylation of both ERα and the mitochondria biogenesis transcription factor NRF1 [67, 238]. The metabolic plasticity of tumors can be also ascribed to the different dependence on UPR^mt^ proteins. Tumor types that are more incline to electrons transport unbalance generate high levels of ROS which determine the activation of UPR^mt^ and the chaperone-mediated recruitment of the cytosolic misfolded proteins by the mitochondria. Targeting such protein recruitment with a new combined therapy could reduce UPR^mt^. In this regard, many clinical studies are investigating new potential inhibitors of UPR^mt^ even in combination with chaperone-targeting compounds, to reduce tumor progression and drug resistance phenomena [239, 240].

### Mitophagy in cancer

Mitophagy plays multiple roles in cancer development and progression [241, 242]. It can serve as a tumor suppressor, maintaining the balance between mitochondria amount/activity by removing damaged or dysfunctional mitochondria in certain cancer subtypes [243, 244]. Mitophagy also reduces ROS formation and limits tumour-initiating step mediated by ROS. However, mitophagy can also drive drug resistance, preventing chemotherapy-induced apoptosis during tumour progression [241, 245]. Functional mitophagy inhibits the accumulation of damaged mitochondria and selectively removes them through autophagosomes preventing carcinogenesis. PINK1 is accumulated in dysfunctional mitochondria activating the recruitment of the E3 ubiquitin protein ligase, Parkin [246–248]. Many studies showed that the activation of PINK1/Parkin pathway can induce mitophagy in cancer cells, removing dysfunctional mitochondria and reducing ROS formation [249]. On the other hand, the deletion or mutation of regulators genes, such as the Parkin family genes, can inhibit mitophagy, promoting carcinogenesis [243]. CSCs are regulated by the mitophagy process as well [225, 250, 251]. Indeed, mitophagy represents a pro-survival pathway for CSCs formation [252, 253], maintaining the stemness and self-renewal ability of CSCs. The inhibition of mitophagy downregulates the expression of essential nuclear genes, such as NANOG, involved in CSCs survival [254, 255].

The inhibition of specific mitogen-activated protein (MAP) kinase signaling pathways can reduce stemness through BNIP3 and BNIP3L activation. The MAPK7 (called also ERK5) inhibitor XMD8-92, suppressed CSCs formation in lung adenocarcinoma and glioblastoma [256]. Recently, a subpopulation of ATP-high cancer cells was identified in breast cancer, which are phenotypically aggressive showing an increased proliferation, stemness, cell migration, invasion and drug resistance, as well as high antioxidant capacity [257–259]. These results demonstrate a difference in OXPHOS regulation ad ATP production between normal and cancer cells. Indeed, the γ-subunit of the mitochondrial ATP-synthase (ATP5F1C) expression is increased in lymph-node metastases compared to primary tumors and is highly over-expressed in distant metastatic lesions [260], and can be targeted by the FDA-approved Bedaquiline in triple negative breast cancer cells [260, 261].

### Mitochondria as pharmacological/therapeutic target in cancer

In the last few decades, the so called “Warburg effect” [262–264], by which cells predominantly use glucose that can be metabolized through glycolysis with generation of lactate in the cytoplasm, or by further metabolism of pyruvate via TCA cycle and OXPHOS [263, 265], has changed based on new evidence showing that mitochondria are not completely dysfunctional in cancer, but can produce similar or increased levels of TCA and fatty acid oxidation intermediates compared to non-tumorigenic cells [266, 267]. OXPHOS might continue its function even while aerobic glycolysis is increased, becoming a protagonist pathway in cancer development. Indeed, in response to oncogenic signals or even to drug therapy, mitochondria biogenesis and activity are upregulated, promoting increased TCA cycle intermediates that sustain metabolic demands of cancer cells [208, 268]. Both glycolysis and TCA are used by cells, although typically one pathway dominates. Cells within a given tumour are predominantly glycolytic, whereas others prefer an OXPHOS metabolic phenotype depending on nutrient availability [269, 270]. Many studies have demonstrated that tumor cells are vulnerable to the inhibition of OXPHOS suggesting that mitochondria float between differing metabolic status [271, 272]. It is now well documented that “metabolic rewiring” is necessary to meet the challenges of high energy demand requested by aggressive tumors for rapid cell division, migration and metastasis formation indicating that mitochondrial energy reprogramming also regulates oncogenic pathways and post-translational modifications of oncoproteins [273]. In the “reverse Warburg effect”, cancer cells induce oxidative stress by inducing aerobic glycolysis neighbour fibroblasts, which in turn secrete pyruvate and lactate taken up by cancer cells to augment mitochondrial OXPHOS [274–276]. This mechanism sheds light on previously unappreciated metabolic heterogeneity and plasticity of cancer cells [277, 278]. Experimental evidence demonstrates that PGC1, a key transcription factor that is regulated by MYC which drives mitochondrial biogenesis and fission, is highly expressed and is poor prognostic marker in numerous cancer types; increasing OXPHOS and establishing drug resistance [206, 279]. PGC1 inhibitors reduce the invasive migratory phenotype of cancer cells by altering the mitochondrial metabolism and the ATP content of the cancer cells [280, 281]. The upregulation of PGC1α induce overexpression of OXPHOS gene set, by providing a structural basis for enhanced OXPHOS in multiple myeloma cells; SR18292, a new PGC1α inhibitor, exerts potent antimyeloma effects becoming a new therapeutic strategy [281]. SR18292 showed efficacy also in pediatric acute myeloid leukemia cells, where PGC1α inhibition decreased the mitochondrial DNA (mtDNA) copy number. PGC1α contributes to enhanced mtDNA copy number predicting disease aggressiveness and poor survival outcome [282]. The mtDNA transferred by non-cancer cells to cancer cells lack functional mitochondria and increases tumour efficacy in animal models [283–285]. Moreover, breast-cancer cells with high telomerase transcriptional activity and stemcell-like phenotypes showed increased mitochondrial mass and membrane potentials [286, 287]. Indeed, the mitochondrial encoded proteins are critically important for cancer aggressiveness; interfering with them determine anticancer effects [288, 289]. CSCs have been shown to rely on OXPHOS instead on glycolysis [251, 290], depending by mitochondrial metabolism for CSCs features maintenance. New or repurposing drugs that target mitochondria and inhibit OXPHOS have been studied as potential anticancer agents [250, 291, 292]. Atovaquone and Metformin, which interfere with OXPHOS by inhibiting Q-cytochrome c oxidoreductase (complex III) and NADH-coenzyme Q oxidoreductase (complex I), respectively, showed a clear efficacy against breast cancer progression and CSCs formation in vitro and in vivo [293, 294].

Preclinical studies in pancreatic and ovarian cancer showed that Atovaquone reduced cancer formation and dissemination in vivo [295, 296] acting as platinum sensitizing agent [297]. Metformin reduces gluconeogenesis in the liver, and is an approved drug for the treatment of type 2 diabetes mellitus [298], demonstrating anti-proliferative activity in vitro and in vivo, and showing anti-cancer effects in the context of clinical trials [299, 300]. Metformin has been shown to affect CSCs that are more dependent by OXPHOS than non-staminal tumor cells [301, 302]. Metformin as complex I inhibitor, is being assessed in over 100 ongoing clinical trials in nondiabetic patients with cancer [303, 304].

Interfering with ATP synthase (complex V), or its ecto-α-subunit [305]), interrupts the electron chain transport and ATP formation. A previously mentioned drug Bedaquiline, targets the mitochondrial ATP-synthase, leading to mitochondrial dysfunction and ATP depletion. Indeed, by affecting the F0-F1 ATP-synthase and in particular the ATP5F1C protein, Bedaquiline showed anticancer activity, reducing CSCs formation, inhibiting migration, invasion and metastasis formation in vitro and in vivo [260, 261]. Mitochondrial metabolic enzymes offer a variety of attractive targets for anticancer treatment [306, 307]. Isocitrate dehydrogenase (IDH) enzymes catalyse the conversion of isocitrate into α-ketoglutarate, which also has roles in cellular processes such as the hypoxic response and gene expression modulation. IDH1 and IDH2 mutants promote oncometabolite formation by disruption of canonical α-ketoglutarate production [308, 309]. Targeting the IDH mutants is a new promising target therapy to prevent glioma or acute myeloid leukaemia (AML) generated by IDH-mutated clones [310–312]. The mitochondria inner membrane transporters, also known as solute carrier family SLC25, are a class of proteins that regulate the traffic between mitochondria and cytosol [313, 314]. Recent studies have shown the involvement of mitochondria membrane transporter in the regulation of cancer plasticity and adaptability. Indeed, many SLC25 transporters are involved in phenomena of drug resistance and cancer failure therapy [315, 316]. Recently, the SLC25A8 protein called mitochondrial uncoupling protein 2 (UCP2), inducing proton leak and involved in the transport of C4 metabolites out of mitochondria [317, 318], has been demonstrated to support growth of KRAS mutated pancreatic cancer [319].

## Anticancer therapies and cardiotoxicity: a role for mitochondria

Adverse cardiovascular events are frequently experienced in cancer patients as a direct consequence of the neoplasm or as a side effect induced by treatments. With the formulation of more advanced anti-cancer regimens, the global improvement of prognostic parameters, and the development of better tools for early diagnosis, an overall prolongment of survival has been observed; on the other hand, cardiovascular side effects may manifest latently, even decades after the completion of the anti-cancer therapeutic scheme. The urge to address this relevant clinical issue has led to deepen the knowledge on the molecular, biochemical, and biological mechanisms of cardiac injury by anti-cancer drugs. Among the multiple obstacles encountered, the wide portfolio of available pharmacological strategies in cancer therapies implies that different mechanisms could converge on the establishment of cardiac toxicity. While this general assumption suggests categorizing cardiac side effects on the basis of the drug class, it should be noted that a unifying perspective is retrieved when looking at mitochondrial aberrations as the main triggers for cardiac damage. Therefore, mitochondria could represent suitable targets of pharmacological interventions aimed at preserving cardiac function in cancer patients (Fig. 3).

A high degree of mitochondrial impairment triggers irreversible cardiac injury, which is usually caused by traditional chemotherapies; these agents dramatically affect the ultrastructural organization of cardiomyocytes, with large vacuolized areas, cytoskeleton disarrays and mitochondrial morphological aberrations; in contrast, newly developed and tailored-cut pharmacological agents induce lower cardiomyocytes damage consistent with benign ultrastructural changes, and overall fewer cardiac side effects.

### Anticancer therapy-induced cardiotoxicity

#### Chemotherapy: the paradigmatic example of doxorubicin

For certain tumors, which are unresponsive to more advanced therapies, chemotherapy still represents the mainstay of treatment. Although it represents an irreplaceable option in some cases, the use of certain chemotherapy agents may determine cardiac damage, particularly for anthracyclines [320, 321]. This class of drugs, which elicit potent antibiotic activity, also exhibits well-acknowledged anti-cancer properties in breast, urogenital, brain and stomach cancers as well as in leukemia, lymphomas, and sarcomas [322–325]. Despite their effectiveness in halting cancer progression, left ventricular alterations, cardiomyopathy, and heart failure are included within the spectrum of possible cardiac manifestations in response to anthracyclines treatment [326–330].

Doxorubicin represents the prototypical and most frequently prescribed member of anthracyclines. Its anti-cancer mechanism relies on the ability to interfere with DNA replication and transcription at multiple levels [331–333]. Cardiovascular and dose-dependent side effects of doxorubicin include hypotension, tachycardia, and arrythmias [334], whereas the most serious cardiac events are represented by cardiomyopathy and congestive heart failure [12, 327, 335–337].

At the molecular level, it has been demonstrated that Doxorubicin enters mitochondria due to its cationic nature, and accumulates within the inner membrane, where it binds to the phospholipid cardiolipin [338]. Not only cardiolipin plays a role in mitochondrial membrane structure, but it regulates the activity and function of diverse mitochondrial proteins, including the enzyme complexes of the electron transport chain [339], determining an increased generation of oxidative stress and a diminished production of ATP [340, 341]. In turn, the impairment of mitochondrial respiration and fatty acid oxidation paves the way for a metabolic reprograming toward the glycolytic pathway [342], possibly anticipating heart failure. The transcriptional profiling of mitochondria-related genes revealed a substantial decline in the expression of genes controlling oxidative phosphorylation and fatty acid metabolism upon Doxorubicin exposure [343]. Additionally, it was shown that the perturbations of energy metabolism reflect a shift toward protein catabolism to obtain fuels [344]. Likewise, Doxorubicin suppresses AMPK activity [345], simultaneously lowering ATP consumption [346].

Together with alterations in functionality, a dramatic reduction in mitochondrial mass is observed upon treatment with Doxorubicin, which is known to activate mitophagy [347] and inhibit mitochondrial biogenesis [348]. Furthermore, ROS accumulation within mitochondria paves the way to mPTP opening and apoptosis [340, 349], further contributing to cardiomyocytes loss. Additional studies have demonstrated that administration of Doxorubicin interferes with the natural antioxidant defense response [350]. In line with this evidence, Zeng and colleagues (2020) show that, in mouse and human cardiac tissues, Doxorubicin increased the expression of NOX1 and NOX4 and induced mitochondrial fission through DRP1 activation, DRP1 translocation into mitochondria with an increase in DRP1 phosphorylation at Ser616 (p-Ser616) and a decrease in phosphorylation at Ser637 (p-Ser637). Along with the ability of Doxorubicin to impair cardiac mitochondrial function decreasing the expression of MFN1, MFN2, and OPA1, and increasing Drp1, activating autophagy and mitophagy in vivo, additional evidence indicates that this anticancer drug can also sensitize the propensity for mPTP to open, inducing apoptosis and necroptosis [351]. Doxorubicin can dose-dependently stimulate mPTP, as a consequence of mitochondrial oxidative damage and Ca^2+^ overload (*i.e.,* both potent inducers of mPTP) typically involved in its acute, sub-chronic, and chronic cardiotoxic actions. Indeed, a recent study reported, in NRVMs, adult mouse ventricular myocytes (AMVMs), and in mice, that inhibiting monoamine oxidase (MAO)-dependent ROS generation abolishes Doxorubicin-induced oxidative stress that generates lipid peroxidation, alteration in mitochondrial membrane potential, mPTP, and redox potential, abnormalities of mitochondrial distribution and perimeter, sarcomere organization, and intracellular Ca^2+^ homeostasis, indicating that mitochondrial ROS formation represents an upstream event of Doxorubicin-induced cardiotoxicity [352]. Moreover, mitochondria isolated from Doxorubicin-treated human atrial trabeculae displayed reduced transmembrane potential and Ca^2+^ retention capacity, while the immune-suppressant cyclosporin A, a blocker of mPTP opening, prevented mitochondrial dysfunction and impaired contractile performance induced by Doxorubicin [349]. This finding was confirmed in vivo, where cyclosporine A mitigates the alteration of mitochondrial bioenergetics and mitochondrial fragmentation secondary to Doxorubicin exposure [353]. On the other hand, Doxorubicin has been shown to stimulate the receptor-interacting protein 3 (RIPK3)-induced activation of Ca^2+^-calmodulin-dependent protein kinase (CaMKII), triggering mPTP opening, thus inducing myocardial necroptosis [354], clearly supporting the role of mPTP opening in Doxorubicin cardiotoxicity in both experimental models and human hearts.

Non-anthracyclines drugs have been shown to trigger cardiovascular side effects [355] also through the alteration of mitochondrial activity and function. This is the case for cisplatin, an alkylating agent used alone or combination with other antineoplastic agents to treat diverse types of tumors [356]. By prompting organelles’ structural and ultrastructural modifications and alterations of ΔΨm, together enhanced ROS generation, cisplatin elicits cumulative cardiotoxicity, which is frequently associated with remarkable nephrological side effects [357]). Among the alkylating agents, high doses of cyclophosphamide and ifosfamide are known to induce acute myopericarditis and severe arrhythmias, which appear to be associated also with the increase of oxidative stress, alterations of IMM, impairment of mitochondrial transport and oxidation of long chain fatty acids, as well as aberrant mitochondrial respiration [358–360].

5-fluorouracile belongs to the class of antimetabolites. Through the inhibition of thymidylate synthase and the impairment of DNA replication and RNA synthesis, 5-fluorouracile elicits antineoplastic action in a broad spectrum of cancers [361]. However, cardiac side effects have been reported [362]. Despite the pathogenetic mechanisms have not been fully clarified, several studies have demonstrated that the drug-induced cardiotoxicity may be associated at least in part to the increased formation of ROS resulting from lipid peroxidation and depletion of glutathione pool, ultimately leading to apoptosis [363]. Furthermore, the accumulation of the highly toxic intermediate fluoroacetate from the biotransformation of 5-fluorouracil may impact on mitochondrial function by inhibiting the Krebs cycle [364].

Other antineoplastic agents with reported cardiovascular toxicity include the antibiotics mitoxantrone (inhibitor of type II topoisomerase), mitomycin C (inhibitor of DNA synthesis and cross-linker) and bleomycin (inhibitor of DNA biosynthesis through the intercalation into DNA helix). One of the most common and shared pathogenetic mechanism of these chemotherapeutics is the induction of oxidative stress, associated with mitochondrial dysfunctions [355, 365, 366]. For instance, mitoxantrone is active in certain solid tumors (metastatic breast cancer and metastatic hormone-refractory prostate cancer), as well as in myeloid leukemia, non-Hodgkin lymphoma and acute lymphoblastic leukemia [367]. The irreversible cardiomyopathy observed after long-term treatment with mitoxantrone is associated with remarkable metabolic impairments that are consistent with aberrant mitochondrial activity, such as decreased ATP levels, alterations of mitochondrial membrane potential, inhibition of ATP synthase expression, together with dysfunction of complexes IV and V [365, 368].

Arrhythmias and conduction disorders have been observed in a small percentage of patients undergoing systemic treatment with the antimicrotubule agent paclitaxel [369]; while the involvement of mitochondrial damage in these side effects has been postulated, drug-induced alterations within the Purkinje fibers and histamine release are more likely to be responsible for the chronotropic actions of paclitaxel [370, 371]

In addition, the vinca alkaloids vincristine and vinblastine, that block microtubule formation in mitotic spindle leading to the arrest of cell division of metaphase, may induce hypertension, angina, and ischemic heart disease [372]. Among the mechanisms proposed, Herradón and co-workers found that alterations of TNFα, eNOS and iNOS in cardiac tissue after chronic treatment with vincristine may be responsible for alterations of heart function; moreover, the increase in mitochondrial connexin 43 expression induced by vincristine suggests that this important cardioprotective regulator counteracts the detrimental increase in free radical to protect the heart tissue from apoptotic damage [372, 373].

#### Radiotherapy

Despite the enormous advances in the clinical management of cancer patients, radiation therapy is still currently indicated in more than 50% of cases, both in the neoadjuvant and adjuvant setting [374, 375]. However, radiation-induced heart disease (RIDH) includes arrhythmias, valvular dysfunction, ischemic heart disease, non-ischemic cardiomyopathy, and pericardial disease [376].

The first evidence that radiation induces relevant subcellular morphological changes in mitochondria was provided more than 50 years ago by means of electron microscopy, which showed a high degree of swelling, disorganization and clumping of the cristae, together with fusion of the inner and outer membranes [377]. mtDNA is a highly susceptible target of ionizing radiation, as the ability to promote single and double strand breaks, nucleotide damage and cross-link, and gene deletion, is not efficiently counteracted by an efficient DNA repair system [378, 379]; in addition, the lack of histones further facilitate the damaging action of radiation on mtDNA [380].

As a consequence of the alterations detected in mitochondrial structure and in mtDNA, a reduced functionality of these organelles is expected, together with an increase in ROS generation. Likewise, irradiated cardiac mitochondria exhibit lower respiratory capacity, consistent with alterations in respiratory chain proteins [381]. A comprehensive proteomic analysis of C57BL/6 mice performed after total body exposure to radiation revealed molecular responses of inflammation, antioxidant defense and cytoskeleton perturbations [382], with mitochondrial proteins being the most strikingly affected by ionizing radiation [382].

#### Targeted therapies

Opposite to chemo/radiotherapy, targeted therapies recognize specific molecular targets implicated in tumor progression, thus warranting higher specificity and fewer overall side effects. However, certain agents belonging to this class exhibit cardiovascular toxicity which may be occasionally critic, particularly when drug combinations are administered, rather than monotherapy regimens [383, 384].

The most frequently evidenced cardiac side effects of targeted therapies include hypertension, left ventricular dysfunction, thromboembolism, and heart failure [383]. Trastuzumab, an antibody targeting the Human epidermal growth factor receptor 2 (HER2), Trastuzumab may trigger an asymptomatic decreased in LV ejection fraction, or in some cases, congestive heart failure [385]. It should be mentioned that Trastuzumab is mostly reversible with the termination of the therapy [386].

Considering the importance of receptors tyrosine kinase (RTKs) in cancer, small-molecule drugs have been designed with the aim to target the ATP binding pockets of TKs, thus hindering the access to the kinase site within the receptor molecule and the subsequent activation of the stimulatory transduction cascade. Despite RTK inhibitors are quite well tolerated at the cardiovascular levels, imatinib and sunitinib are two representative examples of on-target [387] and off-target [388] cardiotoxicity, respectively. Imatinib allows to control chronic myeloid leukemia (CML) and certain gastrointestinal tumors [389, 390]. The drug targets specifically BCR-ABL, a fusion product which activates stimulatory cascades involved in cell proliferation and survival like RAS, PI3K/AKT and STAT5 [390]. As BCR-ABL aberrations occurs in more than 90% of chronic myeloid leukemic cells, the drug is highly specific and effective. Cardiac side events are rarely detected after use of imatinib, totalling on average 1% of the patients receiving the treatment [391]; however, a strict follow up is required for patients with a prior history of cardiovascular disease.

#### Immunotherapy

The wise manipulation of the immune system toward the instigation of an efficient anti-cancer response is the main objective of immunotherapies. In cancer patients the immune system is highly dysregulated, as tumor cells are enabled with peculiar mechanisms to evade immunosurveillance [392]. The impaired recognition of tumor cells sets up the stage for a tolerogenic environment that facilitates disease progression. In recent years, striking breakthrough in the field of clinical immunotherapy have revolutionized anti-cancer approaches [393], as targeting the immune system rather than the tumor cells only, now represents a feasible and successful therapeutic strategy. Immunotherapeutic agents are highly heterogeneous: the modification of patients’ own dendritic-cells (DCs) and T-cells and their subsequent reinfusion has led to the development of DC-based and T-cells based anti-cancer vaccines [394, 395]; in addition, monoclonal antibodies, immune checkpoint inhibitors (ICIs), cytokines and interleukins, together with bi-specific T-cell engagers are included among the portfolio of available anti-cancer immunotherapies. Despite the enormous clinical success obtained using these approaches in certain types of tumors, a long-term assessment of the potential side effects in response to these strategies is still missing, due to their relatively recent clinical implementation. On the other hand, cardiovascular side effects have been reported after administration of ICIs. This class of agents includes monoclonal antibodies directed at the checkpoint inhibitors CLTA4, PD1 and PDL1, a system of receptors and ligands that suppresses T cells activation and inhibits T cell function, thus protecting from an aberrant immune activation [396, 397]. ICIs are able to block the immune checkpoints, thus relieving their inhibitory effect on the immune system, ultimately leading to an efficient anti-cancer immune response. Few cases of myocarditis, HF and impairment of normal hemodynamics have been observed upon administration of ICIs [398–400]. Despite the incidence of cardiovascular toxicity is not currently high, the percentage of patients affected by cardiac disorders is expected to increase in the upcoming years, when more individuals will likely be treated with ICIs.

The mechanisms implicated in the cardiotoxicity of ICIs are far from being fully elucidated, however, it is clear that the PD1/PDL1 signalling is essential in cardiomyocytes immune homeostasis, as it prevents aberrant T-cells activation [401], hence blocking this pathway may elicit detrimental effects like the formation of autoantibodies. In addition, dysregulated immune cells may target normal cardiomyocytes components like cardiolipin, thus compromising cell activity and function [398]. Moreover, several changes in the immune microenvironment may be detected upon administration of ICIs, including imbalanced Treg and Teff cells, abnormal release of cytokines, induction of macrophages polarization and T-cell infiltration [399]. Not surprisingly, immune inflammation, ROS accumulation and mitochondrial impairment may represent key effectors of ICI-induced cardiotoxicity [402, 403].

### Mitochondria-based approaches to relieve the cardiotoxicity of anti-cancer therapies

Considering the crucial role played by mitochondria in the cardiotoxicity of onco-therapies, the pharmacological preservation of mitochondrial function represents a feasible and suitable option for cardioprotective purposes. Therefore, it is not surprising that several anti-oxidant agents may elicit beneficial effects by relieving ROS accumulation and preventing the subsequent mitochondrial damage. In this regard, dexrazoxane is among the most investigated drug for preventing Doxorubicin-induced cardiotoxicity, approved by both FDA and European Medicines Agency (EMA) [404]. After being hydrolyzed into its active compound, dexrazoxane acts as an iron chelator, thereby preventing the formation of peroxide radicals and alleviating oxidative stress. In fact, mitochondrial structure and function appeared preserved in cardiomyocytes stimulated with Doxorubicin in the presence of dexrazoxane, despite certain alterations in mtDNA content were still evidenced [404]. Similarly, other FDA-approved drugs have been shown to decrease the oxidant load and thereby obliterate Doxorubicin-induced cardiotoxicity. This is the case for carvedilol, a β-blocker and vasodilating agent used in the treatment of systemic hypertension, left ventricular dysfunctions and congestive heart failure [405]. By reducing oxidative stress and lipid oxidation, carvedilol has been shown to contrast Doxorubicin-induced cardiac events [405, 406], whereas the drug was ineffective in hampering the cardiotoxicity of other anti-cancer molecules like trastuzumab [407].

Additional agents, of both natural and synthetic origin, were found to reduce ROS production and mitochondrial impairment by doxorubicin, thus promoting cardioprotection; among these, the plant-derived compound Ginsenoside Rh2 [408], the flavonoid Luteolin-7-o-β-d-glucopyranoside [409], the garlic derivative allicin [410], the antihyperlipidemic agent probucol [411], the adiponectin agonist ADP355 [412], the Phosphodiesterase-5 inhibitor sildenafil [413], the alternate G-protein estrogen receptor agonist G-1 [414] and the lipid-lowering statin Fluvastatin [415]. Due to its anti-oxidant properties, vitamin E was also suggested to prevent cardiac damage, while increasing doxorubicin anti-cancer efficacy [416, 417]; however, a recent thorough analysis of trials and systemic reviews highlights certain controversies on the benefit on vitamin E against Dox-induced cardiotoxicity; therefore, further investigations are warranted to turn the inconclusiveness of these findings [418].

Interestingly, Beak and collaborators found that the α1 adrenergic agonist Dabuzalgron, an orally bioavailable and well-tolerated drug previously tested for the treatment of urinary incontinence, efficiently enhances contractile function and restores energy production in Doxorubicin-treated mice [419]; these effects rely on the restoration of mitochondrial function, as evidenced by the increased expression of PGC1α implicated in mitochondrial biogenesis, and other mitochondrial genes regulators of energetic metabolism [419].

Mitochondrial transfer may be used as an efficient tool to hamper doxorubicin-dependent negative effects in the heart. More specifically, the Authors employed patient-specific induced pluripotent stem cell-derived cardiomyocytes (iCMs), which were subjected to doxorubicin injury and then co-cultured with extracellular vesicles (EVs) from mesenchymal stem cells (MSCs). Interestingly, large EVs enriched in mitochondria protected iCMs from injury, whereas small EVs, poorly enriched in mitochondria, failed in their protective effect [420]. iCMs stimulated with mitochondria-enriched EVs exhibited improved contractility, ATP production and mitochondrial biogenesis, whereas a mitochondrial inhibitor (1-methyl-4-phenylpyridinium) hampered this effect [420]. This interesting study sheds new light into the potential of stem cells and mitochondrial transfer as a useful novel approach to preserve cardiac function in doxorubicin-treated cancer patients.

Undoubtedly, mitochondria-preserving strategies could thwart also the toxicity of targeted therapies. In this regard, data from the MANTICORE-101 study (Multidisciplinary Approach to Novel Therapies in Cardiology Oncology Research) have clarified the effects of the angiotensin-converting enzyme (ACE) inhibitor perindopril versus the β-blocker bisprolol in patients with early diagnosis of HER2 positive breast cancer undergoing treatment with trastuzumab [421]. While both drugs were well-tolerated and provided protection against therapy-induced decline in LVEF, an inhibition of left ventricular remodeling was not achieved [421]. However, Shirmard et al. demonstrated that the impairment of mitochondrial aberration is sufficient to protect cardiac function in male adult Wistar rats treated with trastuzumab in combination with curcumin, chrysin and thymoquinone [422].

Likewise, manipulating mitochondrial dynamics could represent a feasible strategy to counteract trastuzumab cardiotoxicity. For instance, the transmembrane protein Connexin-43 (Cx43), if localized at the mitochondrial membranes, regulates these organelles’ structure and function in terms of volume and respiratory capability [423]. By controlling mitochondrial calcium homeostasis and ROS generation, Cx43 is engaged in cardiomyocytes mitochondria to prompt apoptotic effects upon trastuzumab administration [424]; hence, controlling Cx43 recruitment at mitochondria could circumvent trastuzumab-dependent cardiomyocytes death.

Furthermore, the antidiabetic drug metformin, which regulates energy metabolism mainly through the activation of AMPK, ameliorates trastuzumab dysfunctions possibly restoring the energetic capacity of cardiomyocytes [425]; this action could involve both the increased uptake of glucose mediated by insulin, together with the correction of energetic imbalances [425].

Extending these findings, a retrospective analysis performed in a large cohort of almost 7000 women affected by early breast cancer revealed that administration of metformin, but not thiazolidinediones, reduces the risk of cardiac events in response to adjuvant radiotherapy, thus suggesting that administration of AMPK regulators might be a useful post-surgery strategy when radiations are required [426].

It should be mentioned that the sensitivity or resistance to cardiac damage induced by radiations may depend on the myocardial expression of certain inherited variants of mitochondrial genes, thus suggesting that complex genetic modifiers may impact on patients’ cardiac tolerance to radiotherapy [427].

Furthermore, it has been shown that overexpression of certain protective factors like G protein-coupled receptor kinase 2 (GRK2) protects mitochondria from radiation-induced cardiomyocytes injury, whereas silencing GRK2 triggers mitochondrial aberrations suggestive of damage, such as reduced membrane potential together with structural and functional impairments [428]. Considering the evidence that GRK2 interacts with MFN1 and MFN2 to regulate mitochondrial dynamics of fission and fusion [428, 429], the role of GRK2 in regulating mitochondrial function in stressful conditions and the potential of this protein to serve as pharmacological target of intervention in cardioprotective strategies should be explored more in depth. Table 1 summarizes the cardiovascular or anticancer effect of drugs targeting mitochondrial functions.

**Table 1:** Cardiovascular or anticancer effect of drugs targeting mitochondrial functions

Drug	Effect	Mechanism	References
Elamipretide	Normalizes mitochondrial dynamics in failing heart	Water-soluble tetrapeptide that aggregates cardiolipin in the IMM	[156]
Epigallocatechin gallate	Protects the heart from IRI	Inhibits OMA1	[166]
Oligomycin	Protects the heart from IRI Preclinical trials to potentially treat many type of cancers	Stimulates UPR^mt^ Inhibits the F_0_ subunit of mitochondrial ATP-synthase	[167] [436]
Doxycycline	Protects the heart from IRI Down-regulates DNA-PK and mitochondrial biogenesis	Stimulates UPR^mt^ Radiosensitizes tumor initiating cells	[167] [437, 438]
Bedaquiline	Inhibits in vivo metastasis in triple negative breast cancer Preclinical trials to potentially treat many type of cancers	Binding to the c subunit rotor and subunit ε of the ATP synthase Inhibits the γ-subunit of the mitochondrial ATP-synthase (ATP5F1C)	[260, 261] [439]
4-bromo-resveratrol, nicotinamide, sirtinol, cambinol, salermide	Reduce proliferation and progression of many cancers	Sirtuins inhibitors	[440, 441]
Dexrazoxane	Prevents doxorubicin-induced cardiac toxicity	Reduces oxidative stress in mitochondria by chelating iron	[404]
Carvedilol	Prevents doxorubicin-induced cardiac toxicity	Reduces oxidative stress and lipid accumulation in mitochondria	[405, 406]
Gingenoside rh2	Prevents doxorubicin-induced cardiac toxicity	Reduces oxidative stress in mitochondria	[408]
Luteolin-7-o-β-d-glucopyranoside	Prevents doxorubicin-induced cardiac toxicity	Reduces oxidative stress in mitochondria	[409]
Allicin	Prevents doxorubicin-induced cardiac toxicity	Reduces oxidative stress in mitochondria	[410]
Probucol	Prevents doxorubicin-induced cardiac toxicity	Reduces oxidative stress in mitochondria	[411]
ADP355	Prevents doxorubicin-induced cardiac toxicity	Reduces oxidative stress in mitochondria	[412]
Sildenafil	Prevents doxorubicin-induced cardiac toxicity	Reduces oxidative stress in mitochondria	[413]
Fluvastatin	Prevents doxorubicin-induced cardiac toxicity	Reduces oxidative stress in mitochondria	[415]
Dabuzalgron	Prevents doxorubicin-induced cardiac toxicity	Stimulates mitochondrial biogenesis by increasing PGC1α expression	[419]
Metformin	Reduces trastuzumab-induced cardiac toxicity	Enhances cardiomyocyte metabolism (to be confirmed)	[425, 426]

## Conclusions and future perspectives

A large body of evidence indicates that cancer and CVD share numerous pathophysiological mechanisms, such as neuro‐hormonal activation, inflammation, oxidative stress, and immune system alteration, as well as diverse risk factors, including hypertension, obesity, smoking and air pollution, and cardiometabolic disease. The enhanced survival of cancer patients achieved thanks to the progress of screening programmes, cancer therapies, and diagnostic approaches has determined an increase of CVD, including, but not limited to, atherosclerosis and atrial fibrillation. Similarly, an augmented prevention, specific dietary regimens, together with combined pharmacological regimens, improved ventricular assist devices and early percutaneous cardiac intervention, have led to improved prognosis and decreased mortality for HF and other CVD, while determining a simultaneous increase of cancer‐related mortality. On the other hand, different approaches for treating cancer have been associated with cardiovascular complications, including coronary artery disease and HF, conduction defects, and valvular dysfunction [3, 430]. This is the case of radiation exposure (especially to the thoracic region), used in the treatment of various types of cancers, several classes of cytotoxic chemotherapy, displaying potent anticancer activity while causing cardiotoxicity, cardiomyopathy, and vascular toxicity. Also monoclonal antibodies, such as bevacizumab, or tyrosine kinase inhibition (sunitinib, pazopanib, and sorafenib) can be responsible for increased arterial thrombotic events, including myocardial infarction [12, 14, 431]

The complexity of different interacting pathways, that drive both cancer and CVDs and determine the risk of CVDs in cancer patients, as well as the risk of cancer in patients presenting with CVDs, makes it difficult the identification of specific drugs that, through counteracting cardiovascular risk factors promoting cancer development, should reduce both cardiovascular‐ and cancer-related mortality. Therefore, comprehensive and detailed mechanistic studies are needed to further elucidate the molecular determinants driving cancer and CVDs and their common pathophysiological features.

Over the last years there has been increasing focus on the role of mitochondrial dysfunction, in terms of alteration in mitochondrial dynamics and quality control (i.e., key mechanisms to maintain the optimal performance of mitochondria) and on how these processes can participate to cancer development and CVDs progression. Even if the pathophysiological complexity underlying cancer and CVDs cannot be addressed by a single pharmacological approach designed for preventing or resolving mitochondrial damage and dysfunction, improving our understanding of mitochondria-dependent mechanisms in cancer and CVDs may become a useful option to develop additional potential therapeutic targets for the treatment of CVDs and/or cancer. However, if targeting dysfunctional mitochondria for therapeutic purposes proved to be an effective strategy for treating CVDs in pre-clinical studies, the clinical translation of mitochondrial targeting agents (MTAs) has hitherto failed due to several pitfalls that mainly concern pharmacodynamic and pharmacokinetic issues [432]. For instance, blocking mPTP with cyclosporine A, which successfully interfered with rodent models of CVDs [433], is therapeutically unfeasible in cardiovascular patients due to the potent immunosuppressant effect of this drug [432]. Novel cyclosporine A analogues that lack immunosuppressant activity, such as Debio025 and NIM811, are currently under investigation. The efficacy of untargeted antioxidant strategies to attenuate ROS overproduction in mitochondria is also limited by their off-targeted effects on cytosolic ROS, which stimulate cellular survival and other primary functions at moderate levels [432, 434]. It has been suggested to exploit the highly hyperpolarized ΔΨm to specifically deliver cationic antioxidant molecules into the mitochondrial matrix [435]; unfortunately, the ΔΨm is rather depolarized in many CVDs [432]. Moreover, systemically administered MTAs could target the mitochondria that are abundantly located in other cellular compartments, such as brain, skeletal muscle, and vascular endothelium [432]. This limitation could, however, be circumvented by incorporating the MTAs within poly(lactic *co*glycolic acid) (PLGA)-decorated nanoparticles to specifically target the myocardium [432].

Therefore, the quest for more specific and less toxic therapies, aimed at improving therapies efficacy and reducing adverse drug events, still remains an important issue and an open challenge in the field of cardio-oncology.

## Data Availability

Not applicable.

## Abbreviations

ATF: Activating transcription factor
BNIP3: Bcl-2 interacting protein 3
CHF: Chronic heart failure
CMEC: Cardiac microvascular endothelial cells
CML: Chronic myeloid leukemia
CSCs: Cancer stem cells
CVDs: Cardiovascular diseases;
Cx43: Connexin-43
DCM: Dilated cardiomyopathy
DRP1: Dynamin-related protein 1
ER: Estrogen receptor
EVs: Extracellular vesicles
FIS1: Mitochondrial fission protein 1
GRK2 G: Protein-coupled receptor kinase 2
HAoSMCs: Aortic smooth muscle cells
HER2: Human epidermal growth factor receptor 2
HF: Heart failure
HIF-1α: Hypoxia-inducible factor-1α
I/R: Ischemia/reperfusion
ICMs: Induced pluripotent stem cell-derived cardiomyocytes
IMM: Inner mitochondrial membrane
IRI: I/R injury
ISR: Integrated stress response
MASM7: Mitofusin activator small molecules 7
MTAs: Mitochondrial targeting agents
Mdivi-1: Mitochondrial division inhibitor-1
MFF: Mitochondrial fission factor
MFI8: Mitochondrial fusion inhibitor 8
Mfn1: Mitofusin 1
Mfn2: Mitofusin 2
mPTP: Mitochondrial permeability transition pore
mtDNA: Mitochondrial DNA
NRVMs: Neonatal rat ventricular myocytes
OMM: Outer mitochondrial membrane
OPA1: Optic atrophy 1
OXPHOS: Oxidative phosphorylation
PGC-1α: Peroxisome proliferator-activated receptor gamma coactivator 1-alpha
PINK1: PTEN-induced putative kinase 1
RIPK3: Receptor-interacting serine/threonine kinase 3
ROS: Reactive oxygen species
SR: Sarcoplasmic reticulum
TNF-α: Tumor necrosis factor-α
TRAF2: Tumor necrosis factor receptor-associated factor-2
UCP2: Uncoupling protein 2
UPR^mt^: Mitochondrial unfolded protein response
UPS: Ubiquitin proteasome system
VDAC: Voltage-dependent anion channel
ΔΨm: Mitochondrial membrane potential
